# Teaching multimodal LLMs to comprehend 12-lead electrocardiographic images

**DOI:** 10.1038/s41746-026-02551-3

**Published:** 2026-03-16

**Authors:** Ruoqi Liu, Yuelin Bai, Xiang Yue, Ping Zhang

**Affiliations:** 1 Department of Computer Science and Engineering, The Ohio State University, Columbus, OH USA; 2 Shenzhen Institutes of Advanced Technology (SIAT), CAS, Shenzhen, China; 3 Language Technologies Institute, Carnegie Mellon University, Pittsburgh, PA USA; 4 Department of Biomedical Informatics, The Ohio State University, Columbus, OH USA; 5 Translational Data Analytics Institute, The Ohio State University, Columbus, OH USA

**Keywords:** Cardiology, Computational biology and bioinformatics, Engineering, Health care

## Abstract

Electrocardiograms (ECGs) are essential, non-invasive diagnostic tools for assessing cardiac conditions. Existing methods often have limited generalizability, focus on narrow condition sets, and rely on raw physiological signals, which may be unavailable in resource-limited settings where only printed or digital ECG images are accessible. Recent advances in multimodal large language models (MLLMs) offer new opportunities, yet ECG image interpretation remains challenging due to the lack of instruction-tuning data and standardized benchmarks. To address these gaps, we introduce ECGInstruct, the first large-scale ECG image instruction-tuning dataset with over one million samples, covering diverse tasks including feature recognition, rhythm analysis, morphology assessment, and clinical report generation. We develop PULSE, a fully open-source MLLM for ECG image interpretation trained on ECGInstruct. We further curate ECGBench, a human expert-developed benchmark spanning four core ECG interpretation tasks across nine datasets, incorporating both synthesized and real-world ECG images to enable clinically realistic evaluation. Our experiments demonstrate that PULSEestablishes a new state of the art, outperforming general-purpose MLLMs by 21% to 33% in average accuracy. These results highlight the potential of PULSEto improve ECG image interpretation in clinical practice. All code, data and models are available at https://aimedlab.github.io/PULSE/.

## Introduction

Electrocardiograms (ECGs) are essential, non-invasive tools for diagnosing cardiovascular diseases. Despite the availability of automated ECG diagnosis machine learning models^1–3^, their clinical adoption remains challenging. Many models can only classify a restricted set of conditions^2^, limiting their ability to detect previously unseen abnormalities. Additionally, they rely on time-series physiological signals, which may be unavailable in resource-limited settings^4^ where ECGs are stored only as *printed or digital images*^5,6^.

Recent advances in multimodal large language models (MLLMs)^7–10^ have demonstrated exceptional capabilities in vision-language tasks, opening new possibilities for ECG interpretation directly from ECG images (i.e., standard 12-lead ECG visualizations), which are the primary format used by clinicians^11^.

However, adapting MLLMs for ECG image analysis presents several obstacles. First, there are no large-scale ECG image datasets, as most existing ECG datasets contain only raw signal data, necessitating the creation of ECG images. Second, instruction-tuning (i.e., training models using instruction and response examples to follow clinically meaningful ECG-related queries) is underexplored for ECG images, requiring the development of high-quality instruction-response pairs tailored to ECG interpretation. Lastly, the absence of a standardized benchmark for evaluating MLLM performance on ECG images makes it difficult to quantify progress and identify areas for improvement.

**Fig. 1:**
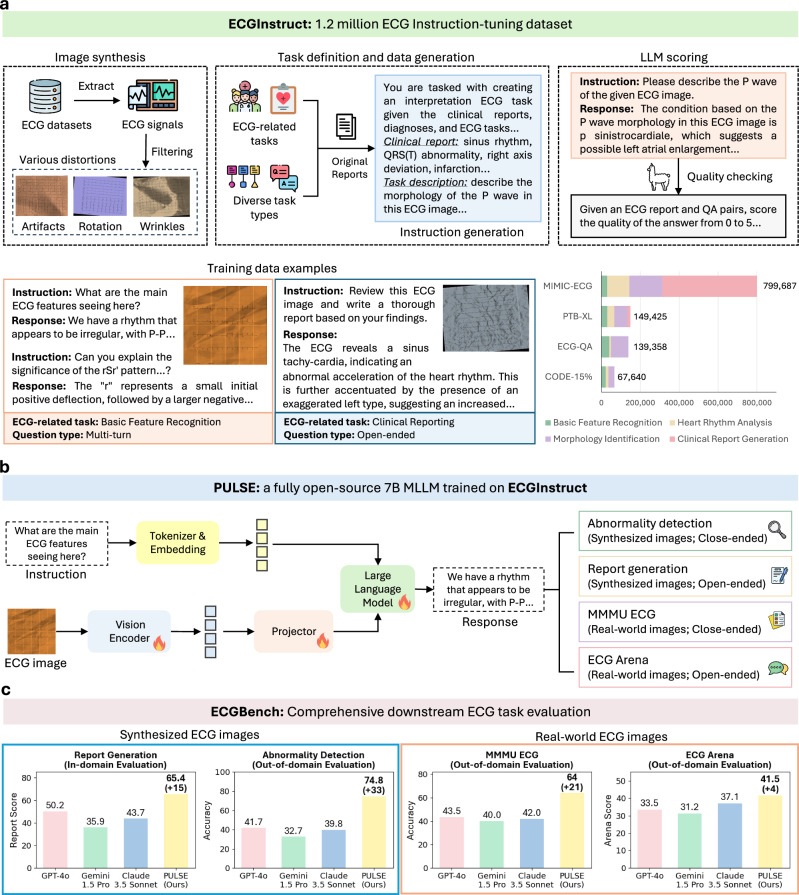
Overview of the ECGInstruct dataset, the PULSE model, and the ECGBench evaluation benchmark. **a** ECGInstruct, a comprehensive ECG image instruction-tuning dataset comprising over one million carefully curated image-text pairs spanning diverse ECG-related diagnostic and reasoning tasks. The ECG images are first synthesized from raw signals with realistic distortions. ECGInstructis curated from clinician-defined tasks, diagnoses, and clinical reports. An independent LLM scores the data for quality control. **b** PULSE, a fully open-source 7B MLLM, interprets ECG images directly for diverse ECG tasks. The model architecture consists of a vision encoder, an LLM, and a projector that aligns visual and textual modalities. **c** ECGBench, an expert-curated benchmark covering comprehensive interpretation tasks and real-world collected ECG images. PULSEoutperforms existing MLLM models by 21–33% accuracy gains in out-of-domain evaluation. Figure created with PowerPoint and Matplotlib.

**Fig. 2:**
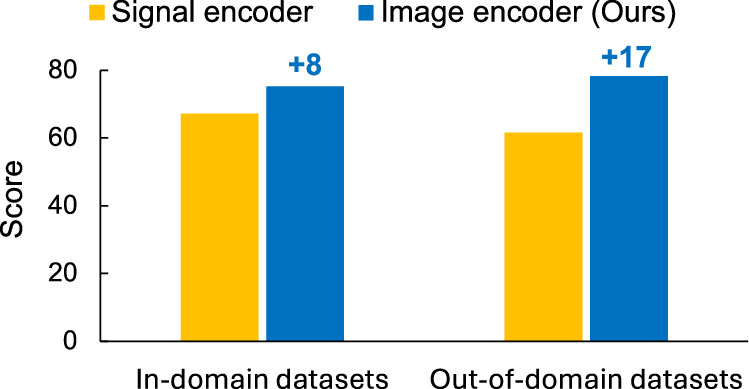
Performance comparison between signal-based encoder and image-based encoder. Figure created with PowerPoint.

To address these challenges, we introduce a comprehensive suite of resources aimed at advancing ECG image interpretation: (1) ECGInstruct, the first large-scale ECG image instruction-tuning dataset with over one million ECG image-text samples (Fig. 1a); (2) PULSE, a fully open-source MLLM with 7 billion parameters trained on ECGInstructthat achieves state-of-the-art performance in ECG diagnosis (Fig. 1b); and (3) ECGBench, a human expert-curated benchmark covering four ECG image interpretation tasks across nine different datasets (Fig. 1c and Fig. 4).

Specifically, ECGInstructintegrates diverse ECG-related tasks informed by clinical expertise, ensuring real-world applicability. To enhance robustness, we introduce synthetic ECG images with common artifacts, helping the model generalize to noisy clinical data. We collect ECG samples from multiple geographic regions to promote adaptability across different populations and healthcare systems. Additionally, we employ a large-scale synthetic data generation pipeline that leverages LLMs for instruction-tuning, with strict quality control through expert validation and automated assessment. Based on the LLaVA architecture^10^, we train PULSE-7B on ECGInstruct, demonstrating that this straightforward training recipe significantly enhances ECG interpretation across various tasks.

For evaluation, we develop ECGBench, a comprehensive benchmark co-designed with clinical experts to assess ECG image interpretation rigorously. Crucially, ECGBenchincorporates real-world ECG images alongside synthetic data, enabling evaluation under clinically realistic conditions. ECGBenchincludes four key tasks: (1) Abnormality detection, aligning diagnostic labels across heterogeneous datasets; (2) Report generation, standardizing expert-validated clinical reports; (3) MMMU ECG, a high-quality multi-choice question set with strict quality control; and (4) ECG Arena, a multi-turn interaction task that simulates real-world reasoning. Our evaluation strategy combines traditional metrics like AUC and F1 with GPT-4o LLM-as-judge scoring^12^ for open-ended tasks, ensuring both objectivity and clinical relevance.

Evaluated on ECGBench, our fully open-source PULSEsets a new state-of-the-art, significantly outperforming proprietary MLLMs across all benchmarks with an average accuracy gain of 21% to 33% compared to GPT-4o on out-of-domain datasets. Ablation experiments demonstrate the importance of incorporating diverse data sources and ECG instruction tasks into the training data. A case study and discussion further illustrate the model’s effectiveness in ECG image interpretation.

## Results

### Baselines and setup

In order to evaluate the performance of our proposed model, we compare it against a set of established methods, including domain-specific methods and state-of-the-art MLLMs.

- Domain-specific methods. We consider six domain-specific methods, including five signal-based methods: METS^13^, MERL^14^, ST-MEM^15^, MMCL^16^, MOMENT^17^; one image-based method: ECG-GPT^18^. These methods are designed for automated ECG diagnosis and typically focus on classifying a restricted set of predefined cardiac conditions.
- Proprietary MLLMs. We consider three proprietary MLLMs: GPT-4o^19^, GPT-4o mini^19^, Gemini 1.5 Pro^20^, and Claude 3.5 Sonnet^21^.
- Open-source MLLMs. We select various open-source models to ensure coverage across different model sizes and visual components, including the general models LLaVA-1.5^22,23^, LLaVA-1.6^10^, Phi-3-Vision^24^, Idefics2-8B^25^, DeepSeek-Vl-7B^26^, Mantis-8B-siglip-Llama3^27^, MiniCPM-V-2.6^28^, InternVL2^29,30^ and state-of-the-art multimodal models LLaVA-OneVision^8^, Qwen2-VL^31^, and the domain-specific models LLaVA-Med^9^.

To avoid data leakage, we explicitly distinguish between in-domain and out-of-domain datasets throughout training and evaluation. ECGInstructis used exclusively for training and validation. For in-domain evaluation datasets (PTB-XL Super, PTB-XL Report, CODE-15%, ECG-QA), a dedicated subset is strictly held out for testing, with no overlap with training samples. All out-of-domain datasets (CPSC 2018, CSN, G12EC, MMMU ECG, ECG Arena) are entirely unseen during training and are used solely for evaluation.

### Evaluation metrics

For abnormality detection, we utilize multi-label classification metrics, including Macro AUC, Macro F1, and Hamming Loss, to evaluate PTB-XL Super, CODE-15%, and CPSC 2018, where multiple correct labels may exist. For the ECG-QA, CSN, and G12EC datasets, we adopt accuracy as the evaluation metric.

For report generation, rather than relying on traditional text generation metrics, we leverage strong LLMs as evaluators, following the approach of ref. ^12^. This method provides a more nuanced evaluation by focusing on key aspects of the reports. Specifically, we use GPT-4o to compare the model-generated reports against those written by cardiologists. We introduce a “Report Perfect Score”, which is based on three critical components of a generated report: (1) Rhythms (0 to 10 points), (2) Waveform Morphology (0 to 10 points), and (3) Diagnosis (0 to 10 points). The final score is the average of these three components, scaled to a maximum of 100 points. The prompt used to query GPT-4o for evaluating the report score is provided in Supplementary Fig. 9.

For MMMU ECG, we adopt accuracy as the primary metric. We have designed systematic, rule-based evaluation pipelines to ensure robust and consistent scoring. To mitigate the potential influence of any intermediate generations (e.g., reasoning steps) in long responses, we employ robust regular expressions and develop response-processing workflows. These are used to extract answer options from the long responses for accurate answer matching. In cases where no valid answer can be extracted from the model’s response, we perform random selection to assign a score.

For ECG Arena, we also employ a strong judge model, GPT-4o, to assess model performance by comparing generated responses with ground truth answers. The evaluation considers three perspectives, each scored on a scale of 0–10: Accuracy (how closely the model’s response matches the ground truth), Completeness (whether the model provides a comprehensive answer covering all aspects of ECG interpretation), and Instruction Adherence (how well the model follows the specific instructions in the question). We calculate the final score by averaging these three aspects and scaling to a maximum of 100 points. The specific prompt used for GPT-4 evaluation is provided in Supplementary Fig. 10.

### Overall performance

We show in-domain and out-of-domain results in Table 1 and 2, respectively. Overall, we observe that PULSEachieves state-of-the-art performance on different datasets and tasks.

**Table 1:** In-domain evaluation results

Datasets	PTB-XL Super			PTB-XL Report	CODE-15%			ECG-QA
Metric	AUC	F1	HL	Report Score	AUC	F1	HL	Accuracy
Random	50.3	33.2	50.1	0	48.8	15.0	32.1	16.2
Domain-specific Methods								
METS	-	65.7^a^	-	N/A	-	-	-	N/A
MERL	74.2^a^	-	-	N/A	-	-	-	N/A
ST-MEM	71.4^a^	-	-	N/A	-	-	-	N/A
MMCL	81.6	-	-	N/A	-	-	-	N/A
MOMENT	**83.3**	-	-	N/A	-	-	-	N/A
ECG-GPT	69.5^b^	53.9^b^	20.1^b^	47.8^b^	68.9^b^	40.1^b^	17.4^b^	N/A
Proprietary MLLMs								
GPT-4o	55.6	28.3	26.2	50.2	59.9	24.9	15.7	35.2
GPT-4o mini	52.0	20.4	31.7	37.1	57.5	22.0	15.1	14.9
Gemini 1.5 Pro	50.7	15.3	27.9	35.9	56.7	20.0	15.9	33.2
Claude 3.5 Sonnet	54.0	27.5	29.6	43.7	58.3	20.3	17.8	34.2
Open-source MLLMs								
LLaVA-Med	50.0	12.3	28.1	24.3	69.2	27.0	33.4	29.5
LLaVA-1.6-34B	50.2	19.9	36.0	17.0	57.2	12.8	16.6	22.4
LLaVA-OneVision-7B	49.8	11.4	34.5	30.0	58.7	17.0	20.6	20.4
LLaVA-OneVision-72B	50.6	29.6	50.4	40.6	52.3	7.0	13.1	25.0
Deepseek-VL-Chat-7B	50.9	15.7	27.9	15.6	63.7	27.5	22.4	21.1
MiniCPM-V-2.6	49.0	37.7	63.8	15.4	56.6	25.3	22.0	20.8
Phi-3-Vision-128k-Instruct	50.0	29.6	48.4	20.2	69.6	22.6	38.8	28.4
Qwen2-VL-72B	54.0	28.3	30.2	48.9	60.6	23.6	16.1	23.7
InternVL2-8B	50.6	14.3	27.8	38.1	55.8	16.1	17.7	22.3
InternVL2-40B	51.2	18.7	34.6	41.8	56.7	16.2	17.4	18.2
PULSE-7B (Ours)	82.9	**76.9**	**10.2**	**65.4**	**91.7**	**87.0**	**4.6**	**71.6**
Δ over best proprietary MLLM	+27	+49	+16	+15	+32	+62	+11	+36
Δ over best open-source MLLM	+29	+39	+18	+17	+22	+60	+9	+42

^a^indicates results from original papers,

^b^denotes results obtained using the provided online software, N/A indicates methods not applicable or not designed for certain tasks, and - indicates unreported scores in original papers. Bold values indicate the best-performing model overall, while underlined values indicate the best-performing proprietary or open-source MLLM baseline. Results on all baselines are provided in Supplementary Table 3.

**Table 2:** Out-of-domain (OOD) evaluation results

Datasets	CPSC 2018			CSN	G12EC	MMMU ECG	ECG Arena
Metric	AUC	F1	HL	Accuracy	Accuracy	Accuracy	Arena Score
Random	51.2	15.1	28.8	11.6	12.1	24.2	0
Domain-specific Methods							
METS	-	-	-	N/A	N/A	N/A	N/A
MERL	**82.8**^a^	-	-	N/A	N/A	N/A	N/A
ST-MEM	70.4^a^	-	-	N/A	N/A	N/A	N/A
MMCL	52.7	-	-	N/A	N/A	N/A	N/A
MOMENT	50.5	-	-	N/A	N/A	N/A	N/A
ECG-GPT	69.3^b^	44.0^b^	9.9^b^	N/A	N/A	N/A	N/A
Proprietary MLLMs							
GPT-4o	50.9	10.6	18.2	57.5	49.2	43.5	33.5
GPT-4o mini	49.2	11.0	25.5	32.1	33.2	39.5	30.1
Gemini-1.5-Pro	50.1	7.4	20.5	50.5	36.0	40.0	31.2
Claude 3.5 Sonnet	52.8	11.5	18.9	51.5	51.4	42.0	37.1
Open-source MLLMs							
LLaVA-Med	50.0	2.5	20.2	13.8	14.1	27.0	15.9
LLaVA-1.6-34B	49.6	19.3	62.8	44.3	45.9	31.0	17.5
LLaVA-OneVision-7B	49.6	8.0	28.3	23.3	25.7	26.0	22.5
LLaVA-OneVision-72B	51.5	12.8	29.4	44.0	42.6	35.0	15.5
Deepseek-VL-Chat-7B	50.7	6.0	20.0	35.7	32.9	34.5	15.3
MiniCPM-2.6	50.0	18.0	48.4	12.7	19.6	34.5	20.4
Phi-3-Vision-128k-Instruct	50.6	19.0	70.2	14.8	18.4	31.0	11.3
Qwen2-VL-72B	50.7	9.8	18.9	35.5	42.9	35.0	10.3
InternVL2-8B	52.1	8.2	22.2	47.7	37.5	30.0	22.9
InternVL2-40B	52.4	8.2	21.4	41.0	45.0	30.5	28.0
PULSE-7B (Ours)	80.7	**65.4**	**6.8**	**87.9**	**81.4**	**64.0**	**41.5**
Δ over best proprietary MLLM	+28	+54	+11	+30	+30	+21	+4
Δ over best open-source MLLM	+28	+46	+12	+40	+36	+26	+14

^a^indicates results from original papers

^b^denotes results obtained using the provided online software, N/A indicates methods not applicable or not designed for certain tasks, and - indicates unreported scores in original papers. Bold values indicate the best-performing model overall, while underlined values indicate the best-performing proprietary or open-source MLLM baseline. Results on all baselines are provided in Supplementary Table 4.

As shown in Table 1, PULSEdemonstrates significant improvements over both proprietary and open-source MLLMs across all in-domain datasets. Specifically, PULSEsurpasses the best proprietary model (GPT-4o) with a 27% improvement in AUC, a 15-point gain in report score, and a 36% increase in accuracy on the PTB-XL Super, PTB-XL Report, and ECG-QA tasks, respectively. Moreover, PULSEachieves notable gains over the best open-source model, with a 29% improvement in AUC, a 17-point gain in report score, and a 42% increase in accuracy on the same tasks. These results highlight the complexity of ECG image interpretation, a task where even the best proprietary models perform near randomly. By fine-tuning on ECGInstruct, PULSEachieves substantial performance improvements, demonstrating the importance of high-quality and task-related instruction-tuning. While some domain-specific models achieve comparable or even superior performance on particular datasets, their highly specialized designs often limit their ability to generalize across diverse tasks, especially open-ended reasoning, and settings where only ECG printout images are available without associated raw signals. This lack of flexibility restricts their broader applicability in real-world clinical environments, where task requirements can vary significantly.

Table 2 presents the comparison results on out-of-domain datasets, where PULSEconsistently delivers outstanding performance. Notably, it achieves a significant 21% improvement in accuracy on the MMMU ECG benchmark compared to GPT-4o. This substantial improvement indicates the PULSE’s robustness and ability to generalize to unseen and real-world ECG images. The ECG Arena benchmark presents a more challenging task for all models, characterized by a multi-turn, open-ended question-answering format, which is designed for real-world ECG images and closely simulates real clinical scenarios. Despite these challenges, PULSEstill surpasses the best proprietary model by 4 points and outperforms the leading open-source model by an impressive 14 points in terms of arena score. These results highlight PULSE’s relative strength in handling complex, clinically-oriented ECG interpretation and analysis. Additionally, the performance gap across models on this challenging benchmark indicates considerable room for future improvements in this task.

### Effect of training data source

Given that ECGInstructis compiled from diverse datasets, it is crucial to examine how each dataset contributes to the model’s overall performance. Table 3 presents a comparative analysis of models trained on various dataset combinations. The model trained exclusively on PTB-XL (P) exhibits the lowest performance across all datasets, indicating the limitations of relying on a single data source for effective generalization. As we progressively incorporate additional datasets into the training set, the model’s performance consistently improves. These results highlight the importance of curating diverse training data, as expanding beyond a single source enhances the model’s capacity to generalize across datasets and tasks. We provide an ablation study on different instruction tasks in Supplementary Table 7.

**Table 3:** Performance of different training dataset combinations

Training Data	PTB-XL Super	PTB-XL Report	CSN	CODE 15%	ECG QA	CPSC	G12	MMMU ECG	ECG Arena	AVG
P	70.3	60.8	85.5	33.1	29.6	31.2	68.6	46.0	31.0	50.7
P + M	76.2	66.5	91.4	50.1	33.6	60.2	82.0	64.5	39.6	62.7
P + M + C	76.2	67.9	90.2	87.4	41.2	58.8	78.7	61.5	42.0	67.1
P + M + C + E	76.9	65.4	87.9	87.0	71.6	65.4	81.4	64.0	41.5	**71.2**

P: PTB-XL, M: MIMIC-IV-ECG, C: CODE-15%, E: ECG-QA. F1 for PTB-XL Super, CODE-15%, and CPSC; Accuracy for CSN, ECG-QA, G12, and MMMU ECG; Report Scores for PTB-XL Report; Arena Scores for ECG Arena. AVG denotes the average across all metrics. Bold values indicate the best-performing model overall.

### Comparison between signal encoder and image encoder

We present the comparison results between the image-based encoder (ours) and the signal-based encoder in Fig. 2. To ensure a fair and controlled comparison, both models are trained end-to-end using the exact same training data, instruction-tuning tasks, optimization settings, and overall model architecture, differing only in the encoder head used to process ECGs. Specifically, the image-based model encodes ECGs as digital images, while the signal-based model encodes ECGs as time-series. We adopt a randomly initialized 1D-ResNet18^14^ that is fully trained during instruction tuning as the ECG signal encoder. The supervision signals (ground-truth responses) for both models are derived from clinician annotations and reports in the underlying datasets used to construct ECGInstruct. The results show that the image-based encoder consistently outperforms the signal-based encoder across different evaluation tasks, with particularly significant improvements observed in out-of-domain datasets. These findings highlight that encoding ECGs as images not only aligns with the goal of enabling broader applicability of automated ECG diagnosis, especially in resource-constrained or remote settings (where only printed or digital ECGs are available) but also empirically surpasses the performance of the signal-based encoder model.

### Case study

**Fig. 3:**
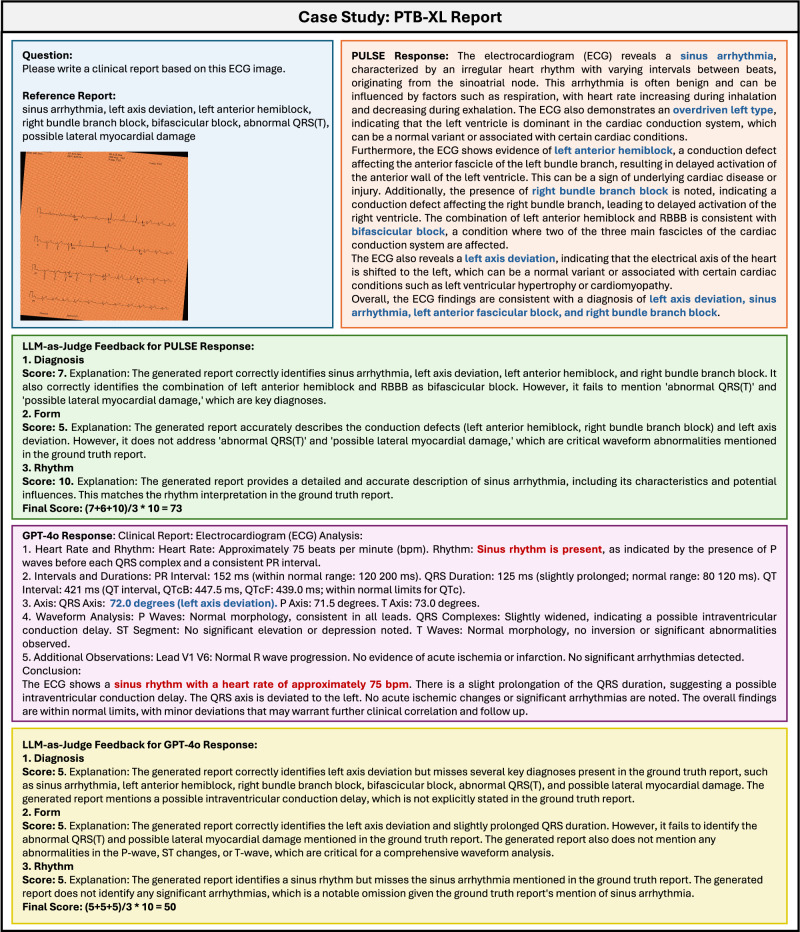
Comparison of model outputs on ECG report generation task (Example 3). Blue indicates correct information, while red highlights errors. Our model’s output mostly aligns with the ground truth report, achieving a report score of 73. In comparison, GPT-4's output partially aligns with the ground truth report. Figure created with PowerPoint.

We present some examples from our benchmark, comparing the outputs of our model with GPT-4o for ECG report generation (Supplementary Fig. 11 and Fig. 12) and ECG Arena (Supplementary Fig. 13). While GPT-4o is capable of generating reports and answering questions by following instructions, it often produces responses that, although well-structured and seemingly relevant, contain significant inaccuracies in interpretation. In contrast, PULSEconsistently provides more accurate responses that align closely with the ground truths. Additionally, we observed that GPT-4o tends to over-rely on its OCR capabilities when textual information (e.g., printed axis labels, numerical values like heart rate or QRS duration) is present in images, leading to superficial reasoning based on text rather than a deep analysis of visual data. As shown in Fig. 3, GPT-4o identifies a left axis deviation based on the printed QRS axis degree, without analyzing the visual waveform patterns. If such axis information were absent, the model would likely fail to identify the deviation.

### Human expert performance

We engaged three clinical experts in cardiology (see Supplementary Table 8 for demographic and professional information) to evaluate a sample of 30 questions from the MMMU ECG. The evaluation was conducted remotely. Each expert received a standardized document containing the ECG images and corresponding multiple-choice questions. Experts were provided with ECG images in both printed and digital formats, and were allowed to zoom in and inspect details as needed for accurate interpretation. They were instructed to complete the task independently without external assistance, relying solely on their clinical knowledge. Their responses were then aggregated to calculate individual and average accuracy.

Table 4 presents the performance comparison. Human experts achieved strong results, with an average accuracy of 84.4%, and the top-performing expert reaching 93.3%. In contrast, PULSEachieved 64.0%, outperforming GPT-4o, which scored 43.5%. These results highlight the difficulty of the task and the value of domain-specific instruction tuning, while also suggesting that further improvements are needed to match expert-level reasoning.

**Table 4:** Comparison results on MMMU ECG among human experts, GPT-4o, and PULSE

Category	MMMU ECG (%)
Human Expert (Low)	70.0
Human Expert (Medium)	90.0
Human Expert (High)	93.3
Human Expert (Average)	84.4
GPT-4o	43.5
PULSE	64.0

## Discussion

In this paper, we study the problem of ECG image interpretation, which is a crucial task in assessing cardiac conditions. We develop PULSE, a fully open-source MLLM trained on ECGInstructwith over one million samples across a diverse range of ECG-related tasks. Evaluated on the proposed benchmark, ECGBench, our model shows state-of-the-art performance, surpassing both proprietary and open-source MLLMs across multiple in-domain and out-of-domain evaluation datasets. This work demonstrates the potential of using MLLMs for enhancing ECG image analysis and interpretation in clinical applications.

Many domain-specific models have been proposed to enhance automatic ECG diagnosis^1–3^. For example, ref. ^2^ applied convolutional neural networks (CNNs) to encode ECG signals for diagnosing 6 types of abnormalities. To reduce dependence on high-quality labeled data, recent studies^13–15^ have further explored self-supervised learning approaches using unlabeled ECG training data. For example, ref. ^14^ proposed an ECG representation learning framework by integrating the ECG signals and clinical reports, showing improved performance in zero-shot ECG classification tasks. Despite these successes, most approaches treat ECG data as temporal physiological signals, which could be limiting in certain resource-constrained or remote settings where only printed or digital images are available. Recently, a few methods^5,6,18^ have been proposed for ECG diagnosis using ECG images. For example, ref. ^18^ developed a diagnostic report generation framework for ECG images, which is built upon a BEiT^32^ vision transformer encoder and a GPT-2^33^ decoder. However, their model is only capable of the clinical report generation task, without generalizability to other diverse tasks. In contrast, our study investigates the capabilities of MLLMs for ECG image interpretation. We curate a diverse range of instruction-tuning datasets to fine-tune the model, thus improving model generalizability.

Recent advancements in MLLMs have shown promising results in various healthcare domains. General medical multimodal models such as LLaVA-Med^8^, MedPaLM^34,35^, and Med-Gemini^36^ have demonstrated capabilities in processing diverse medical data types. Additionally, domain-specific multimodal models have been developed for specialized fields like pathology^37,38^ and radiology^39^. These models have shown potential in integrating visual and textual information to support clinical decision-making and medical education. However, despite the importance of ECG data in cardiac diagnosis and monitoring, current MLLMs often struggle to process ECG images effectively. This limitation highlights a significant gap in the application of MLLMs to cardiology, where the ability to interpret both visual ECG representations and accompanying clinical information is crucial.

Instruction-tuning has proven effective in the multimodal domain, particularly in vision-language models like LLaVA^22^, MiniGPT-4^40^ and InstructBLIP^41^. These models demonstrate impressive generalizability across various visual understanding and reasoning tasks. While multimodal instruction-tuning has been applied to general medical imaging tasks^9,34^, its application to ECG images remains largely unexplored. A recent work^42^ introduced a targeted instruction-tuning framework and fine-tuned existing open-source LLMs for ECG report generation. However, their approach is limited by a single-task instruction dataset focused solely on report generation, potentially constraining its adaptability to other ECG-related tasks. Moreover, their work also treats ECG data as temporal signals, whereas our paper focuses on encoding ECG images with MLLMs, which is more applicable to real scenarios where only printed or digital ECG images are available.

Beyond overall benchmark performance, it is important to assess whether the proposed data synthesis and training paradigm generalizes across different MLLM backbones. To this end, we fine-tune Qwen2-VL-7B using ECGInstructand compare its performance with that of LLaVA-v1.6-Vicuna-7B across multiple tasks, as shown in Supplementary Table 6. While both backbone models demonstrate strong performance, LLaVA consistently outperforms Qwen across all benchmarks, including PTB-XL Super (76.9 vs. 75.1), PTB-XL Report (65.4 vs. 62.8), and MMMU ECG (64.0 vs. 60.4). These results suggest that the proposed training data and methodology are broadly effective.

To further evaluate robustness under realistic acquisition conditions, we assess model performance on photographs of printed ECG images. Specifically, we sample 150 cases from the out-of-domain evaluation datasets (excluding MMMU ECG and ECG Arena, which already contain real paper ECGs), print the images, and capture photographs under varying conditions. As shown in Table 5, the model exhibits only modest performance degradation when applied to photographed printouts, supporting its robustness to common real-world artifacts and its applicability in practical clinical scenarios.

**Table 5:** Performance comparison on digital versus printed ECG images. Bold values indicate the best-performing model overall

Models	CPSC 2018 F1	CPSC 2018 AUC	CSN Accuracy	G12EC Accuracy
Digital images	**59.0**	**75.2**	**96.0**	**81.4**
Printed images	47.8	70.6	92.0	80.0

In addition to model accuracy and robustness, the reliability of the evaluation protocol is critical for interpreting results. To assess the validity of the proposed LLM-based scoring approach, we compared its outputs directly against human evaluation conducted by clinical experts (cardiologists). The expert evaluation results demonstrate that LLM-based scores closely mirror expert clinical judgments across both datasets. As shown in Supplementary Table 9, GPT-4o’s report-generation scores (51.9) and human scores (50.8) yield a Pearson correlation of 0.934, while its ECG Arena scores (32.8 vs. 35.1) correlate at 0.920. Similarly, our model’s report scores (62.8 vs. 64.1) achieve a Pearson correlation of 0.919, and its ECG Arena scores (37.4 vs. 39.0) correlate at 0.917. These consistently high correlation coefficients suggest that the proposed LLM-based metric aligns with human evaluation.

Despite these encouraging results, several limitations remain. While PTB-XL^43^ provides expert-annotated diagnostic information, its original reports are not always fully narrative and may consist of structured key points or multilingual entries. To mitigate this, we systematically rewrite and translate the original annotations into standardized natural-language reports while preserving their clinical meaning (using a prompt as shown in Supplementary Fig. 7) and validate a subset through expert review and consistency checks against SCP-ECG statements. Nevertheless, richer clinician-authored narrative reports would further benefit report generation tasks, and integrating such datasets remains an important direction for future work.

Taken together, these results highlight several clinically relevant use cases enabled by ECG image interpretation. The proposed approach supports scenarios in which raw signal data are unavailable, such as scanned or photographed paper ECGs, legacy medical records, and cross-institutional referrals. Unlike traditional classification models^1–3^ that are limited to predicting a fixed set of labels, our model supports richer forms of clinical interaction, including feature recognition, rhythm analysis, report generation, and multi-turn reasoning. By unifying these capabilities within a single framework, the model aligns more naturally with real-world clinical workflows, allowing users to upload an ECG image together with targeted diagnostic or interpretive queries.

Nevertheless, important challenges remain in translating benchmark performance into real-world clinical deployment. Although ECGBenchcovers a diverse set of clinically motivated tasks, bridging the gap between controlled evaluation and clinical practice will require closer collaboration with clinicians, prospective testing in hospital environments, and task designs that more directly reflect real clinical decision-making processes. Deployment also raises critical safety and bias considerations, including the risk of incorrect or overconfident outputs, dataset biases related to patient demographics or acquisition conditions, and potential misuse without appropriate clinical oversight. Addressing these concerns will require clinician-in-the-loop evaluation, bias-aware dataset curation, and the development of safeguards to support responsible deployment.

Looking ahead, several directions are essential for advancing ECG image-based MLLMs toward clinical readiness. While PULSEdemonstrates strong performance across evaluation datasets, more complex and open-ended tasks remain challenging and demand stronger reasoning and instruction-following capabilities. Progress along these dimensions will likely require advances in data, model design, and evaluation. Future work may incorporate more diverse instruction-following data, as well as scaled high-quality chain-of-thought and multi-turn supervision derived from expert knowledge, structured medical ontologies (e.g., SNOMED CT^44^), clinical guidelines, and medical literature, to better guide intermediate reasoning steps.

Beyond implicit feature learning from images, a promising direction is to incorporate explicit ECG measurements (e.g., intervals, amplitudes, axes) extracted via a calibrated measurement module for scanned/photographed paper ECGs as complementary signals via feature-enhanced instruction-tuning data and joint multimodal training. Integrating such measurement extraction with our MLLM could enable both structured numeric outputs and narrative clinical interpretations that are better grounded in verifiable measurements.

In addition, while this work emphasizes robustness to image-domain artifacts, incorporating explicit signal-domain noise modeling represents an important avenue for future research. Systematic analysis across different noise types and severity levels may help identify failure modes and improve robustness under real-world clinical conditions. Finally, to support long-duration ECG monitoring scenarios, such as Holter recordings and wearable devices, future work will explore hierarchical and temporal aggregation mechanisms that enable event-level reasoning and longitudinal summarization across segmented ECG windows.

## Methods

In this section, we present ECGInstruct, the first large-scale and comprehensive instruction-tuning dataset for ECG image interpretation (Sec.??), and PULSE, a fully open-source 7B MLLM trained on ECGInstruct(Sec.??). We then describe the development of ECGBench, a new benchmark suite designed to evaluate ECG image interpretation across clinically meaningful tasks (Section “ECGBench: A comprehensive benchmark for ECG image interpretation”).

### ECG image synthesis for training and evaluation

Electrocardiography (ECG) is a fundamental diagnostic modality that captures the electrical activity of the heart over time, providing both spatial and temporal insights into cardiac function. A standard ECG recording is represented as a 12-lead multivariate time series, where each lead offers a distinct viewpoint on cardiac electrical activity. The six limb leads (I, II, III, aVR, aVL, and aVF) capture electrical activity in the frontal plane, while the six precordial leads (V1-V6) provide complementary views in the horizontal plane.

To enable large-scale training and evaluation of MLLMs on ECG images, we synthesize realistic ECG image representations from raw signal data using the ECG-image-kit framework^45^. This framework converts digital ECG signals into clinically styled ECG images while allowing controlled introduction of distortions and variations that reflect real-world acquisition, printing, and scanning artifacts commonly observed in clinical practice.

We generate standard 12-lead ECG images following established clinical plotting conventions, including black waveform traces over white backgrounds with red grid lines arranged in a 4 × 3 layout. The synthesized images include standard paper-ECG elements such as calibration pulses (1 mV amplitude and 0.2 s width) and preserve the original waveform samples and inter-lead relationships through a deterministic plotting process. In addition to these canonical renderings, we introduce a diverse set of perturbations to increase visual variability and robustness.

Specifically, we apply augmentations that simulate physical distortions and image quality degradation frequently encountered in paper ECGs. These include wrinkles and creases that mimic wear and tear, as well as random rotations to reflect misaligned scans or prints. To account for heterogeneity in acquisition systems and digitization quality, we vary image resolution, background color (e.g., slight yellowing associated with aging or poor scanning), noise levels, and overall image sharpness. We further introduce variations in aspect ratio, image size, and ECG plot placement within the image to reflect differences across ECG machines, printers, and scanning pipelines. In a small fraction of cases (with probability 0.02), grid lines are removed entirely to model alternative ECG presentation styles.

To further enhance realism, we optionally insert metadata into the image header to simulate annotations commonly present in clinical ECG printouts. For PTB-XL, we extract patient demographics (e.g., age and sex) and basic ECG features (e.g., heart rate and axis deviations) from the PTB-XL+ annotation dataset^46^. These attributes are randomly sampled and rendered onto the synthesized images, increasing visual diversity while providing contextual cues consistent with real-world ECG records.

In addition to the standard 4 × 3 format, we synthesize alternative lead layouts commonly used in clinical practice, including 12 × 1 (single-row), 6 × 2 (two-row), and other widely adopted configurations. These variations expose models to a broad range of ECG presentation styles and reduce overfitting to a single canonical layout.

Overall, the synthesis process is designed to balance realism and diversity. Approximately half of the generated images include augmentations, resulting in an approximate 1:1 ratio between clean and distorted ECG images. This balance ensures that models trained on the synthesized data remain accurate on clean digital ECGs while maintaining robustness to the visual artifacts and variability encountered in real-world clinical settings.

### ECGInstruct: curation of ECG image instruction-tuning dataset

Existing ECG datasets lack ECG images suitable for training multimodal large language models (MLLMs). To address this gap, we curate ECGInstruct, a large-scale instruction-tuning dataset designed to support ECG image understanding and clinical reasoning. Built upon the ECG image synthesis procedure described in Section “ECG image synthesis for training and evaluation”, ECGInstructemphasizes three key properties: (1) realistic ECG image representations that reflect common clinical variability, (2) a diverse set of expert-informed ECG interpretation tasks, and (3) data sourced from geographically distinct populations. A summary of the dataset is provided in Table 6.

**Table 6:** Summary of ECGInstruct

Source Dataset	Task	Type	N
PTB-XL^43^	Feature	Cls, Opn, Fil, MCQ	30K
	Rhythm	Cls, Opn, Fil, MCQ	36K
	Morphology	Cls, Opn, Fil, MCQ	67K
	Report	Opn	16K
ECG-QA^51^	Feature	Cls	40K
	Rhythm	Cls	9K
	Morphology	Cls	90K
MIMIC ECG^47^	Feature	Cls, Opn, Fil, MCQ	29K
	Rhythm	Cls, Opn, Fil, MCQ	115K
	Morphology	Cls, Opn, Fil, MCQ	169K
	Report	Opn	487K
CODE-15%^49^	Feature	Cls	22K
	Rhythm	Cls	14K
	Morphology	Cls	31K
Total (ECGInstruct)			1.2M

Feature: basic feature recognition, Rhythm: heart rhythm analysis, Morphology: morphology and pathology identification, Report: clinical report generation. Cls: close-ended QA, Opn: open-ended QA, Fil: fill-in-the-blank, MCQ: multi-choice QA (See Supplementary Table 1 for full details).

To construct a comprehensive set of ECG-related tasks, we consulted domain experts to curate diverse and clinically relevant tasks covering four different categories. Each category is designed to address specific aspects of ECG interpretation and analysis, including (1) basic feature recognition (see examples in Supplementary Fig. 1), (2) heart rhythm analysis (see examples in Supplementary Fig. 2), (3) morphology and pathology identification (see examples in Supplementary Fig. 3) and (4) clinical report generation (see examples in Supplementary Fig. 4). Basic feature recognition (e.g., interval or segment, etc.) forms the foundation of ECG interpretation, enabling the model to grasp essential cardiac parameters. Heart rhythm analysis (e.g., arrhythmias, conduction abnormalities, etc.) and morphology and pathology identification (e.g., wave shape, pathological conditions, etc.) are more advanced and critical aspects of ECG analysis, ensuring that the model can detect and classify complex conditions accurately. Lastly, clinical report generation mirrors the process of healthcare professionals synthesizing a comprehensive interpretation of an ECG. By incorporating clinical experts’ insights, we encourage the model to learn the practical skills required in a clinical context.

Based on the original diagnoses and clinical reports from the existing ECG datasets, we curate diverse types of tasks, including multi-choice questions, fill-in-the-blank, close-ended QA, and open-ended QA. This variety of task types not only enhances the model’s versatility but also mimics the diverse cognitive processes involved in real-world ECG interpretation. By incorporating these varied task types, we aim to develop a more robust and adaptable model capable of handling a wide spectrum of ECG-related queries and analyses.

To ensure broad applicability and generalizability, we collect publicly available ECG data from four different sources across geographically distinct regions: (1) PTB-XL^43^: a large Germany-based ECG dataset; (2) MIMIC-IV-ECG^47^: a large set of ECGs for patients who appear in the MIMIC-IV Clinical Database from Beth Israel Deaconess Medical Center in Boston^48^; (3) CODE-15%^49^: an ECG dataset from a central ECG repository from Minas Gerais, Brazil under the clinical outcomes in digital electrocardiology (CODE) study^50^; (4) ECG-QA^51^, a question answering dataset for ECGs that is constructed based on PTB-XL^43^. This diverse geographical representation enhances the model’s ability to generalize across different populations and healthcare systems, accounting for potential variations in ECG patterns and interpretations across regions.

To ensure the quality of generated instructions and corresponding responses, we apply an independent LLM as a judge to perform large-scale quality filtering. Importantly, this LLM judge is not used as a ground-truth evaluator of correctness, but as a scalable tool to identify and remove low-quality or inconsistent synthetic pairs, following common practice in large-scale instruction tuning^12,52,53^. This process involves several steps: (1) initial generation: instructions and responses are first generated using our primary model; (2) evaluation criteria: we establish a set of evaluation criteria including the instruction relevance, clarity, answerability of the responses, etc; (3) LLM judge and scoring: an independent LLM (Llama 3^54^) is used as a judge to assess each instruction-response pair against established criteria and assign scores (see prompt in Supplementary Fig. 8); (4) feedback loop: low-scoring items are flagged for human expert review and potential revision or removal; (5) iterative refinement: based on the scoring patterns and human expert input, we continually refine our instruction generation process. By combining automated LLM evaluation with human expert oversight, we create a robust system for maintaining and improving the quality of our instruction-response pairs.

Since large-scale annotation of ECG features is extremely expensive and time-consuming, we develop an automatic data synthesizing pipeline to address this data scarcity issue. We utilized clinical reports from PTB-XL and MIMIC-IV-ECG as initial seed data and leveraged an advanced LLM (i.e., Llama-3-70B-Instruct) for data synthesis. Building upon the expert-in-the-loop process and diverse data resources described in the previous sections, we synthesized a substantial volume of ECG-related instructions and corresponding responses. These were based on expert-provided examples and real-world scenarios, with the specific prompts used in this process detailed in the Supplementary Information. For datasets lacking comprehensive reports, such as CODE-15%, we manually constructed diverse templates to transform the existing data into an instruction-response format. Formally, we have the data synthesis process as follows,

1 $${D}_{s}={F}_{s}({{\mathtt{Prompt}}}_{s}(D);\theta )$$

where *θ* is the teacher LLM (i.e., Llama3-70B-Instruct), *D* is the initial seed data (e.g., clinical reports from PTB-XL), Prompt_*s*_ is the text prompt used to guide the generation, *F*_*s*_ is the quality control function used to shepherd the synthetic data.

### PULSE: model design and training

We develop PULSE, a fully open-source 7B MLLM trained on ECGInstructfor ECG image interpretation. Our model architecture closely follows that of LLaVA^10,23^, adapting it for ECG image analysis. The model architecture contains three core components: a vision encoder, an LLM, and a projection layer that aligns visual and textual modalities. Specifically, we use the CLIP-ViT-Large-Patch14-336 model as the vision encoder to process ECG images, and Vicuna-1.5-7B as the LLM to generate text responses. A lightweight 2-layer multilayer perceptron (MLP) serves as the projector, mapping high-dimensional visual features from the CLIP encoder onto a sequence of image tokens compatible with the LLM’s input space (see Supplementary Table 2 for details of the model parameters).

To enable effective visual grounding, we embed the image representation at the beginning of each dialogue using a special token < image >. These image tokens are generated by extracting visual features from ECG inputs and aligning them with textual tokens. For high-resolution ECG images, we employ the Anyres strategy, which segments images into multiple 336 × 336 sub-images. Features from these segments are concatenated with global image features, enriching the model’s perception across varied ECG scales and formats.

The training data is formatted in a multi-turn chatbot style consistent with the Vicuna-1.5-7B format. Each instance consists of an image, a task-specific instruction, and a target response. The instruction is query or task related to the ECG image, and the target response is the expected output or prediction based on the image and instruction. The image is always positioned at the start of the dialogue, ensuring it serves as the contextual foundation throughout the conversation. An example prompt is: “Human: < image > Describe this ECG image. \n Assistant: This image...”.

We fine-tune all components of the model end-to-end, including the vision encoder (*θ*_enc_), projection layer (*θ*_proj_), and LLM (*θ*_llm_). The model is optimized using an autoregressive objective that maximizes the likelihood of each token in the target response given the instruction, prior tokens, and visual input. Formally, given an instruction **Q**, a target response **A**, and an image **I**, all represented as sequences of tokenized inputs, we train the model by maximizing the likelihood of each token in **A**, indexed by *i* = 1, …, *L* as:

2 $${\mathcal{L}}=-\mathop{\sum }\limits_{i=1}^{L}\log p({{\bf{A}}}_{i}| {{\bf{A}}}_{1:i-1},{\bf{Q}},{\bf{I}};{\theta }_{{\rm{enc}}},{\theta }_{{\rm{proj}}},{\theta }_{{\rm{llm}}})$$

This objective guides the model to generate responses that are both accurate and contextually appropriate by leveraging information from the instruction, prior generated tokens, and the visual features extracted from the ECG image. We train the model over three epochs using a batch size of 128, a learning rate of 2e-5, and a cosine learning rate scheduler with a 5% warm-up phase. Cross-entropy loss is computed over the assistant’s response, guiding the model to generate accurate and coherent outputs grounded in the ECG image and dialogue context. The model is trained on 8 H100 GPUs, each running for 40 hours, totaling 320 GPU hours of computation.

### ECGBench: a comprehensive benchmark for ECG image interpretation

**Fig. 4:**
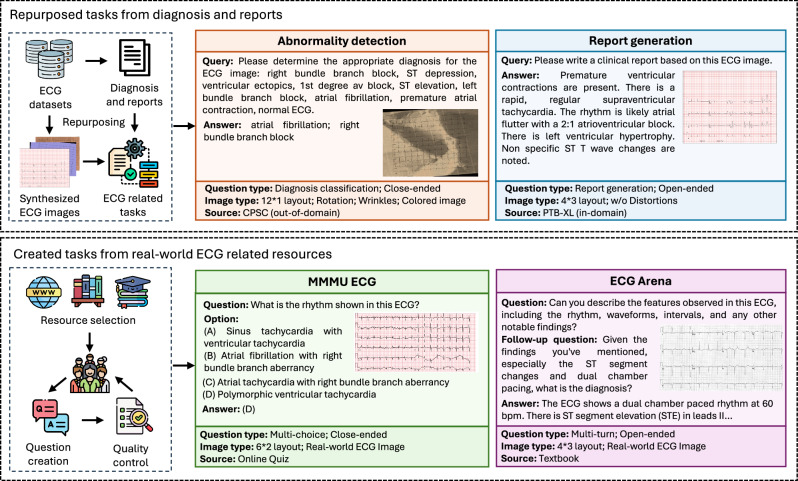
The data curation process for ECGBench. There are four key tasks involved: (1) two repurposed tasks (abnormality detection and report generation) derived from existing ECG datasets, where ECG images are synthesized from raw signals, and queries/answers are extracted based on diagnostic and clinical reports; (2) Two newly developed tasks using external resources, where real-world ECG images and associated questions and answers are collected and generated from real-world sources. Figure created with PowerPoint.

We present ECGBench, a comprehensive benchmark for evaluating MLLMs on ECG image interpretation. Our benchmark contains both repurposed tasks from existing datasets and newly created tasks from external resources. We provide the data curation process in Fig. 4 and details of each evaluation dataset in Table 7.

**Table 7:** Overview of evaluation datasets in ECGBench

Evaluation Dataset	Task	Type	# Samples	Image Source	In-domain?
PTB-XL Super	Abnormality Detection	Close-ended	2082	Synthesized	YES
PTB-XL Report	Report Generation	Open-ended	500	Synthesized	YES
CODE-15%	Abnormality Detection	Close-ended	1400	Synthesized	YES
ECG-QA	Abnormality Detection	Close-ended	1317	Synthesized	YES
CPSC 2018	Abnormality Detection	Close-ended	2061	Synthesized	NO
CSN	Abnormality Detection	MCQ (8-option)	1611	Synthesized	NO
G12EC	Abnormality Detection	MCQ (8-option)	2026	Synthesized	NO
MMMU ECG	Multimodal Understanding	MCQ (4-option)	200	Real-world	NO
ECG Arena	Multi-turn Conversation	Open-ended	50	Real-world	NO

This collection contains both in-domain and out-of-domain problems across four key tasks with diverse answer types.

One core component of ECGBenchfocuses on detecting cardiac abnormalities from ECG images. We curate this task by repurposing six existing ECG datasets: three in-domain datasets: PTB-XL (Super)^43^, CODE-15%^49^, ECG-QA^51^, and three out-of-domain datasets: CPSC 2018^55^, CSN^56,57^ and G12EC^55^. For all datasets, we first synthesize images using raw signals and then curate queries based on the original diagnosis and reports. For datasets with fewer than 10 diagnostic labels, we curate close-ended questions. For those with more labels, we construct multi-choice questions with 8 options, including the original diagnosis and randomly sampled negative labels.

In addition, we evaluate the model’s ability to generate detailed clinical reports from ECG images. For this task, we benchmark using 500 randomly selected reports from the test set of PTB-XL^43^, which contains clinical report annotations curated in routine practice and reviewed by cardiologists. We note that these original annotations may contain uncertainty or noise, so we treat them as reference labels rather than an absolute gold standard.

Inspired by MMMU^58^, a widely adopted evaluation benchmark for MLLMs, we manually curate an ECG version with 200 multi-choice questions with the help of medical school students. The curation process involved three key steps: (1) Resource selection: We gathered ECG materials from multi-source collection of real-world ECG images curated from publicly available educational and clinical resources. These include ECG textbooks, peer-reviewed clinical case reports, and widely used online ECG teaching platforms. The images reflect routine clinical practice at the originating institutions and span diverse cardiac conditions. The images naturally include variations in recording conditions, digitization quality, background artifacts, and baseline disturbances. (2) Question creation and collection: Five medical school students with basic knowledge of ECG were recruited for this task. They extracted existing questions from the collected resources. For ECG images accompanied only by clinical interpretations, the annotators created questions based on these interpretations. Additionally, they formulated new questions drawing from their expertise, ensuring a balance between various ECG interpretation aspects (e.g., rhythm analysis, morphology assessment, clinical interpretation). (3) Quality control: To maintain high standards, we implement a quality control process. In particular, each question was reviewed by at least two other annotators, checking for accuracy and clarity. An independent reviewer cross-checked the final images, questions, and answers against the original sources to ensure fidelity to the source material. Any discrepancies or ambiguities were resolved during this process.

Finally, to assess the model’s instruction-following ability in ECG comprehension, we introduce ECG Arena, inspired by MT-Bench ^12^ and Arena-hard ^53^ used in general LLM chat evaluations. We curate 50 multi-turn ECG-related questions, focusing on open-ended interactions. The data curation process for ECG Arena, like MMMU ECG, involves three main steps: resource selection, question creation, and quality control. The key distinction is that MMMU ECG focuses on multiple-choice questions, whereas ECG Arena involves more complex, flexible multi-turn, open-ended questions. Each follow-up question is contingent on the initial question and its response, making the process more challenging and reflective of real-world applications. Since multi-turn conversations are rare in existing sources, this posed significant challenges during data curation. To address this, annotators created such conversations by referencing original clinical interpretations and ECG images. The questions are designed to feel natural and simulate a real clinical setting (e.g., the first question may ask about basic findings from the image, followed by a question about potential clinical causes or diagnoses based on those findings).

## Supplementary information

Supplementary Information

## Data Availability

All datasets are publicly available: the training data is available at https://huggingface.co/datasets/PULSE-ECG/ECGInstruct, and the evaluation data is available at https://huggingface.co/datasets/PULSE-ECG/ECGBench.

## Code availability

The source code for this paper is available on the GitHub repository at https://github.com/AIMedLab/PULSE. Any additional information required to reanalyze the data reported in this paper is available from the lead contact upon request.
