# Supplementary material for: Teaching multimodal LLMs to comprehend 12-lead electrocardiographic images
Source: NPJ Digit Med. 2026 Mar 16;9:349. doi: 10.1038/s41746-026-02551-3 (PMC13136441; doi:10.1038/s41746-026-02551-3)
Supplement: Supplementary file 1 — Supplementary Information [file 41746_2026_2551_MOESM1_ESM.pdf]

Table of Contents in Supplementary Information

A Details of Instruction-tuning Datasets . . . 30

B Prompts . . . 36

C Main Results . . . 42

D Ablation Study . . . 44

E Human Expert Performance . . . 46

F Human Evaluation . . . 46

G Case Study . . . 47

A Details of Instruction-tuning Datasets

The detailed statistics of curated instruction-tuning datasets are provided in Table 1, with training examples of Figs. 1, 2, 3, 4.

Source Dataset	Task	Type	# Samples
PTB-XL	Basic Feature Recognition	Close-ended QA	22,759
		Open-ended QA	906
		Fill-in-blank	449
		Multi-choice QA	5,716
	Heart Rhythm Analysis	Close-ended QA	19,550
		Open-ended QA	201
		Fill-in-blank	436
		Multi-choice QA	16,179
	Morphology and Pathology Identification	Close-ended QA	50,166
		Open-ended QA	2,649
		Fill-in-blank	680
		Multi-choice QA	13,432
	Clinical Report	Open-ended QA	16,302
PTB-XL Total			149,425
ECG-QA	Basic Feature Recognition	Close-ended QA	40,154
	Heart Rhythm Analysis	Close-ended QA	8,993
	Morphology and Pathology Identification	Close-ended QA	90,211
ECG-QA Total			139,358
MIMIC-ECG	Basic Feature Recognition	Close-ended QA	759
		Open-ended QA	4,759
		Fill-in-blank	6,470
		Multi-choice QA	17,186
	Heart Rhythm Analysis	Close-ended QA	48,625
		Open-ended QA	5,262
		Fill-in-blank	11,487
		Multi-choice QA	49,352
	Morphology and Pathology Identification	Close-ended QA	8,241
		Open-ended QA	81,080
		Fill-in-blank	18,264
		Multi-choice QA	61,456
	Clinical	Open-ended QA	486,746
MIMIC-ECG Total			799,687
CODE-15%	Basic Feature Recognition	Close-ended QA	22,177
	Heart Rhythm Analysis	Close-ended QA	13,893
	Morphology and Pathology Identification	Close-ended QA	31,570
CODE-15% Total			67,640
`ECGInstruct` Total			1,156,110

Supplementary Table 1: Detailed data statistics of `ECGInstruct`.

Training Data Examples: Basic Feature Recognition

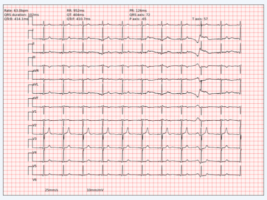

Instruction:
What are the main ECG features I'm seeing here?

Response:
Let's break it down. We have a rhythm that appears to be irregular, with varying P-P intervals. The heart rate is not explicitly stated, but based on the ECG tracing, it seems to be within the normal range. The cardiac axis appears to be normal as well. One notable feature is the presence of an rSr' pattern in lead V1, which is likely a normal variant. Additionally, we can see some nonspecific T wave changes in the septal leads.

Follow-up Instruction:
Can you explain the significance of the rSr' pattern in lead V1? Is it a cause for concern?

Response:
The rSr' pattern in lead V1 is often seen in healthy individuals, especially in younger people. It's considered a normal variant because it doesn't necessarily indicate any underlying cardiac pathology. The "r" represents a small initial positive deflection, followed by a larger negative deflection (the "S" wave), and then a secondary positive deflection (the "r'"). This pattern is thought to be related to the normal activation sequence of the right ventricle. Since it's isolated to lead V1 and doesn't appear to be associated with any other abnormalities, it's unlikely to be a cause for concern in this case.

Sub-task: Basic Waveform Understanding; **Question type:** Open-ended; **Source dataset:** MIMIC-IV-ECG

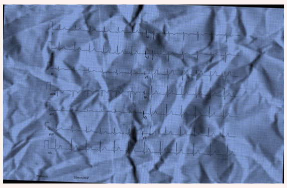

Instruction:
What can be inferred about the heart's electrical conduction system based on this ECG?
Options:
A. There is a block in the electrical conduction pathway
B. There is an abnormality in the heart's electrical conduction system
C. The heart's electrical conduction system is functioning normally
D. There is a rhythm disturbance

Response:
This image indicates that the heart's electrical conduction system is functioning normally, as there are no apparent abnormalities or disturbances in the electrical activity. The presence of a sinus rhythm and a normal axis suggests that the heart's electrical conduction system is operating within normal limits, without any significant defects or anomalies. This ECG image is consistent with a normal heart, and there is no evidence of any conduction system abnormalities.
We can conclude that the answer is C. The heart's electrical conduction system is functioning normally

Sub-task: Conduction System Interpretation; **Question type:** Multi-choice QA; **Source dataset:** PTB-XL

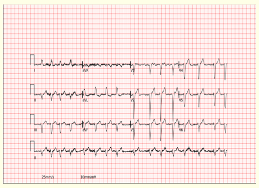

Instruction:
You are reviewing an ECG tracing in a clinical setting. Based on the ECG morphology, fill in the blanks:
This ECG tracing is most consistent with a rhythm of ______________________, with a cardiac axis of ______________________, and a bundle branch block of the __________ bundle.

Response:
This ECG tracing is most consistent with a rhythm of **atrial fibrillation**, with a cardiac axis of **left axis deviation**, and a bundle branch block of the **left** bundle.

Sub-task: Axis and Rhythm Determination; **Question type:** Fill-in-the-blank; **Source dataset:** MIMIC-IV-ECG

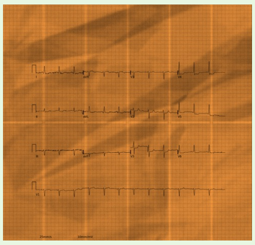

Instruction:
Given this ECG image, please answer the following question. From the provided options, select all that apply. List your selections, separated by slashes.
Question:
Which ECG leads are affected by baseline drift?
Options:
llead III, lead V2, none, lead V4, lead aVL, lead V5, lead V6, lead aVF, lead V3, lead aVR, lead V1, lead I, lead II. Provide the accurate response without additional details.

Response:
lead aVF/lead V3

Sub-task: Lead Placement Determination; **Question type:** Close-ended QA; **Source dataset:** ECG-QA

Supplementary Figure 1: The Examples of basic feature recognition instructions for finetuning PULSE. Figure created with PowerPoint.

Training Data Examples: Heart Rhythm Analysis

Instruction:
Analyze the ECG image and identify the combination of cardiac rhythm abnormalities that can be diagnosed simultaneously in this patient. Rank the abnormalities in order of their potential impact on the patient's hemodynamic stability, with 1 being the most critical. Use a semicolon-separated list with the rhythm abnormality followed by its severity ranking

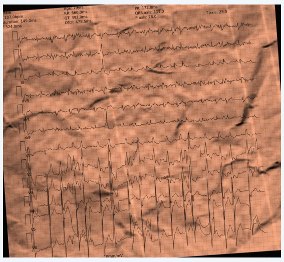

Response:
Sinus tachycardia; 1; Right bundle branch block; 3; Premature atrial contraction; 2; Premature ventricular contraction; 2.
Justification: Sinus tachycardia is ranked as the most critical abnormality since it can lead to decreased cardiac output, increased myocardial oxygen demand, and potentially exacerbate underlying cardiac conditions. Premature atrial contractions and premature ventricular contractions are ranked equally as they can both disrupt normal cardiac function, although their individual impact might be less severe than sinus tachycardia. Right bundle branch block, while still a significant abnormality, is ranked last as it may not have an immediate impact on hemodynamic stability, but rather serves as an indicator of underlying cardiac pathology.

Sub-task: Rhythm Analysis; **Question Type:** Open-ended; **Source Dataset:** PTB-XL

Instruction:
Imagine you're an ER doctor reviewing this ECG, what would be your primary concern regarding the patient's heart rhythm? Directly output one of the following options without explanation:

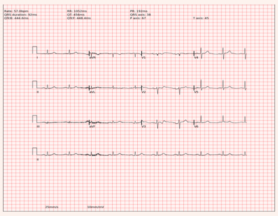

Options:
A) Tachycardia; B) Ventricular Fibrillation; C) Atrial Fibrillation; D) Bradycardia;
E) Long QT Syndrome; F) First-Degree AV Block; G) Second-Degree AV Block;
H) Normal Sinus Rhythm

Response:
D) Bradycardia

Sub-task: Arrhythmia Identification; **Question Type:** Multi-choice QA; **Source Dataset:** MIMIC-IV-ECG

Instruction:
If you were to categorize the ECG rhythm of this patient into one of the following options: Normal Sinus Rhythm, Atrial Fibrillation, Ventricular Tachycardia, or Sinus Rhythm with Premature Atrial Contractions,
which one would you choose? ______________

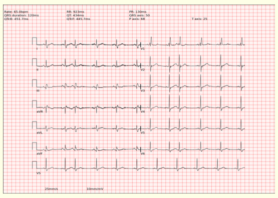

Response:
Sinus Rhythm with Premature Atrial Contractions

Sub-task: Rhythm Classification; **Question Type:** Fill-in-the-blank; **Source Dataset:** PTB-XL

Instruction:
As part of a heart health assessment, your task is to examine the ECG, identify all fitting options from the provided list, and enumerate them using semicolon:
1dAVb(1st degree av block), RBBB(right bundle branch block),
LBBB(left bundle branch block), SB(sinus bradycardia), ST(sinus tachycardia),
AF(atrial fibrillation).
Only answer based on the given options without any explanation.

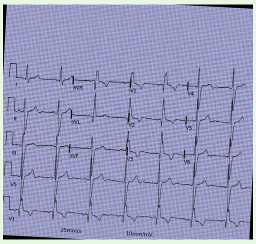

Response:
RBBB(right bundle branch block)

Sub-task: Conduction Abnormality Detection; **Question Type:** Close-ended QA; **Source Dataset:** CODE-15%

Supplementary Figure 2: The Examples of heart rhythm analysis instructions for finetuning PULSE. Figure created with PowerPoint.

Training Data Examples: Morphology and Pathological Condition Identification

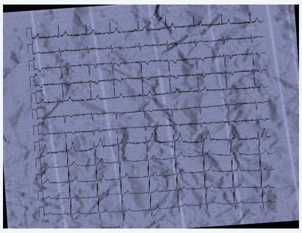

Instruction:
What specific features of the ECG tracing suggest the presence of a right bundle branch block, and how do these features relate to the overall cardiac conduction pattern, considering the morphology of the QRS complexes in leads V1 and V2? Provide a detailed explanation in 2-3 sentences.

Response:
The ECG tracing exhibits a right bundle branch block (RBBB) due to the characteristic wide S waves in leads I and V6, accompanied by QS complexes in leads V2, indicative of delayed right ventricular depolarization. The QRS complexes in lead V1 display a prolonged duration, exceeding 140 ms, with a characteristic "M" or "W" shape, further supporting the diagnosis of RBBB. This conduction pattern is a result of a block in the right bundle branch, leading to an altered sequence of ventricular activation, causing the delayed and abnormal depolarization of the right ventricle.

Sub-task: Wave Morphology Analysis; **Question Type:** Open-ended; **Source Dataset:** PTB-XL

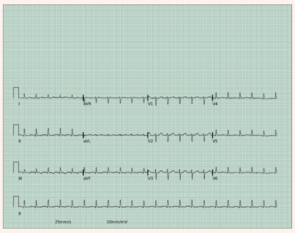

Instruction:
Answer the following multiple choice question. Think step by step before answering.
What is the likely location of a previous myocardial infarction (MI) based on this ECG?

Options:
A. Anterior wall B. Lateral wall C. Inferior wall D. Septal wall

Response:
The ECG shows a pattern consistent with a previous MI, as evidenced by the abnormal Q-waves and ST-segment changes. The location of the MI can be inferred by the leads involved. We can observe that the inferior leads (II, III, and aVF) show the most prominent changes, indicating that the MI likely occurred in the inferior wall. Based on this, we select C. Inferior wall

Sub-task: Interpretation of Ischemia or Infarction; **Question Type:** Multi-choice QA; **Source Dataset:** MIMIC-IV-ECG

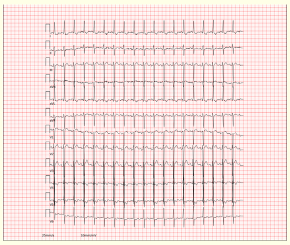

Instruction:
Compare the QRS complex morphology in leads V1-V4 to that of a normal sinus rhythm ECG. Explain the significance of the observed differences, considering the context of intraventricular conduction disorder and delayed R progression.
Then, fill in the blanks: The QRS complex morphology in leads V1-V4 is most indicative of _________. (Provide a detailed explanation in 2-3 sentences before filling in the blank.)

Response:
The QRS complex morphology in leads V1-V4 exhibits a notable difference from that of a normal sinus rhythm ECG. Specifically, the delayed R progression and intraventricular conduction disorder in V4 suggest a disruption in the normal ventricular depolarization sequence, resulting in altered QRS complex morphology. This deviation is significant because it indicates a pathological EKG pattern, which may be associated with underlying cardiac conditions such as left ventricular hypertrophy.
The QRS complex morphology in leads V1-V4 is most indicative of **pathological left ventricular conduction**.

Sub-task: Pathological Conduction Identification; **Question Type:** Fill-in-the-blank; **Source Dataset:** PTB-XL

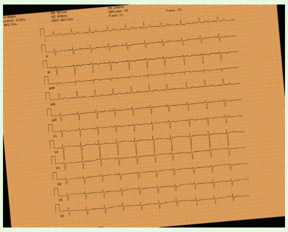

Instruction:
Imagine you are an expert cardiologist reviewing this ECG image. Considering the patient's history and this ECG, what could have contributed to the development of the prolonged QT interval?
Rank your top three possibilities in order of likelihood, using a semicolon-separated list.

Response:
Hypokalemia; certain medications or drugs; genetic mutations or channelopathies.

Sub-task: Electrolyte imbalances Interpretation; **Question Type:** Open-ended QA; **Source Dataset:** PTB-XL

Supplementary Figure 3: The Examples of morphology and pathological condition identification instructions for finetuning PULSE. Figure created with PowerPoint.

Training Data Examples: Clinical Reporting

Instruction:
Review this electrocardiogram image and provide your expert diagnosis.

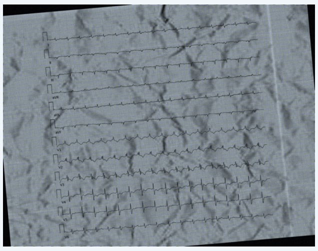

Response:
The electrocardiogram (ECG) reveals a sinus tachycardia, characterized by a heart rate exceeding 100 beats per minute, indicating an abnormal acceleration of the heart rhythm. This is further accentuated by the presence of an exaggerated left type, suggesting an increased electrical activity in the left ventricle.
Additionally, peripheral hypotension is noted, indicating lower-than-normal blood pressure in the peripheral arteries, which may be a concern as it can lead to inadequate perfusion of vital organs.The ECG also demonstrates an incomplete right bundle branch block (RBBB), which suggests a delay or obstruction in the electrical conduction pathway of the right ventricle.
Furthermore, the QRS complex is abnormal, with an altered morphology and duration, indicating a disruption in the ventricular depolarization process. The presence of an old inferior infarct is likely, suggesting a previous myocardial infarction (MI) in the inferior wall of the heart.
Moreover, the ECG shows signs of old ST and T wave abnormalities, indicating possible previous episodes of ischemia or injury to the myocardium. The ECG also suggests likely anterior ischemia or left ventricular strain, which may indicate ongoing or previous damage to the anterior wall of the heart.

Question Type: Open-ended; **Source Dataset:** PTB-XL

Instruction:
Examine the ECG signal image and produce a thorough report based on your findings, endin with the final diagnosis.

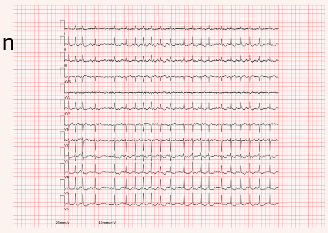

Response:
The electrocardiogram (ECG) reveals atrial fibrillation with a rapid ventricular response, indicating a chaotic and rapid heart rhythm originating from the atria. Additionally, there are signs of a possible anterior myocardial infarction, although the age of the infarct cannot be determined from this ECG. Furthermore, ST-T changes in the inferior and lateral leads suggest myocardial ischemia, which may be partly attributed to the rapid heart rate and rhythm, but also raises concerns for underlying coronary artery disease.
Final Diagnosis: Atrial fibrillation with rapid ventricular response, possible anterior myocardial infarction, and signs of myocardial ischemia.

Question Type: Open-ended; **Source Dataset:** MIMIC-ECG

Supplementary Figure 4: The Examples of clinical reporting instructions for finetuning PULSE. Figure created with PowerPoint.

**Model Parameters**	
Total	7.06B
Vision Encoder(clip-vit-large-patch14-336)	303.5M
Connector	21M
LLM(Vicuna-1.5-7B)	6.74B
**Training Parameters**	
Learning Rate	2e-5
Weight Decay	0.0
Warmup Ratio	0.03
Learning Rate Scheduler	Cosine
Batch Size	128
**Vision Encoder Arch**	
Hidden Size	1024
Input Resolution	336
ViT Layer	24
ViT Heads	16
Patch Size	14
**LLM Arch**	
Hidden Size	4096
Max Context Length	4096
Attention Heads	32
Hidden Layers	32
KV Heads	32

Supplementary Table 2: Summary of model parameters.

B Prompts

The prompt used to synthesize instruction tasks (Fig. 5). The prompt used to synthesize ECG multi-turn dialogue (Fig. 6). The prompt used to revise (and translate) original reports (Fig. 7). The prompt used to score and filter generated instruction data (Fig. 8). The prompt used to evaluate the generated report (Fig. 9). The prompt used to evaluate the ECG Arena (Fig. 10).

Prompt: Multi-task Data Synthesizing

Your task: Create a complex ECG visual task based on the given report and target task type:

Guidelines for task creation:
1. Design a concise yet challenging graduate-level task that requires deep reasoning.
2. Frame the task as interacting with an actual ECG image, without mentioning the report. Make the task visually centric, assuming direct ECG image analysis.
3. Strictly base all information on the given ECG report only. Avoid tasks and answers that are inconsistent with the report.
4. Avoid restating the report or using phrases like "As described in the report."
5. Generate one task from a diverse range of task types, including but not limited to:
   Direct questions (e.g. "What is the heart rhythm?")
   Hypothetical scenarios (e.g. "Imagine you're an ER doctor reviewing this ECG...")
   Comparative tasks (e.g. "How does this ECG differ from a normal sinus rhythm?")
   Explanation requests (e.g. "Explain the significance of the QS complexes seen in V2.")
   Problem-solving scenarios (e.g. "Given these ECG findings, what further tests might you order?")
   Educational prompts (e.g. "Teach a medical student about the key features of this ECG.")
   Role-playing scenarios (e.g. "You're consulting with a cardiologist about this ECG. What do you tell them?")
   Decision-making tasks (e.g. "Based on this ECG, would you clear this patient for surgery? Why or why not?")
6. Specify a clear, appropriate output format within the task instructions(free-form, "think-step-by-step", direct output the short answer(in one phrase or one sentence), JSON format, table, list, different delimiters(such as commas, semicolons, numeric order), etc.). Do not limited to the given task type and format, you have the freedom to design any type of task you deem appropriate.
7. Focus the task on one or more of the following ECG analysis aspects:
   a. Basic ECG feature interpretation (e.g. heart rate, rhythm, cardiac axis)
   b. Diagnosis and classification (e.g. diagnosis identification, waveform classification, rhythm classification)
   c. Waveform and interval analysis (e.g. P wave morphology, PR interval, QT interval, QRS complexes, T wave morphology)
8. Ensure the task complexity aligns with the given report's information.

After creating the task:
1. Provide a detailed, accurate answer to your own task.
2. Ensure your answer is comprehensive and strictly based on the report.
3. Strictly follow the output format and requirements specified in your task instructions.

ECG Report:
{report}

Target Task Type:
{target}

Present your work in this format:
Task: [Concise content of the ECG tasks, including required output format. Do not include phrases like "Output format:..." or like "[Insert image here]", but in more natural expression.]

Response: [Comprehensive response following the task's requirements, strictly based on the report]

Do not include any content outside of the Task and Response sections.

Supplementary Figure 5: The prompt used to synthesize ECG instruction tasks based on clinical reports. Figure created with PowerPoint.

Prompt: Multi-turn Dialogue Synthesizing

Your task: Create a 2-4 turn dialogue between a medical professional and an AI assistant analyzing an ECG, based on the given report:

Guidelines for dialogue creation:
1. Design a series of questions and answers that progressively explore the ECG findings in depth, suitable for graduate-level medical professionals.
2. Frame the dialogue as if the medical professional is directly analyzing an actual ECG image, without mentioning the report. Make the conversation visually centric, assuming direct ECG image analysis.
3. Strictly base all information on the given ECG report only. Avoid including details inconsistent with the report.
4. Do not use phrases like "As described in the report," "The report mentions," or "The term..." The dialogue should not appear to reference an external report.
5. Begin with direct questions about basic ECG features, then progress to more complex interpretations and clinical implications.
6. Include a mix of question types, with an emphasis on direct questions:
 - Direct questions (e.g., "What are the main ECG features?", "What is the heart rhythm?")
 - Requests for explanations (e.g., "Can you explain the significance of the QS complexes?", "What the cause of these features?")
 - Clinical reasoning questions (e.g., "Given these findings, what's your diagnosis?")
 - Hypothetical scenarios (e.g., "How would you manage a patient presenting with this ECG?")
7. Focus the dialogue on one or more of the following ECG analysis aspects:
 a. Basic ECG feature interpretation (e.g., heart rate, rhythm, cardiac axis)
 b. Diagnosis and classification (e.g. diagnosis identification, waveform classification, rhythm classification)
 c. Waveform and interval analysis (e.g. P wave morphology, PR interval, QT interval, QRS complexes, T wave morphology)
 d. Clinical implications and management
8. Ensure the dialogue complexity aligns with the given report's information.

After creating the dialogue:
1. Provide extremely comprehensive and detailed answers from the AI assistant's perspective. Each response should thoroughly cover all relevant aspects of the question asked.
2. Ensure all answers are comprehensive and strictly based on the report, without explicitly referencing it.
3. Make the dialogue flow naturally, as if a real user is progressively exploring the ECG findings.
4. Structure the AI assistant's responses to be highly readable:
 - Break down complex information into digestible parts.
 - Use bullet points or numbered lists to organize information
 - Include brief explanations of medical terms or concepts when necessary
 - Provide context for why certain findings are significant
 Aim for a balance between depth of information and clarity of presentation in each response.

ECG Report:
{report}

Present your work in this format:
Human: [First question about the ECG]

Assistant: [Comprehensive response based strictly on the report]

Human: [Follow-up question delving deeper into the ECG analysis]

Assistant: [Detailed answer providing further insights]

[Continue the dialogue for up to 2 more turns if necessary, ensuring a natural progression of inquiry]

Do not include any content outside of the dialogue format. Ensure that the entire conversation appears to be about analyzing an actual ECG image, without any indication that the information comes from a written report.

Supplementary Figure 6: The prompt used to synthesize ECG multi-turn dialogue as instruction-tuning data. Figure created with PowerPoint.

Prompt: Report Revision

I will provide you with an ECG report. Please expand the report into a comprehensive and detailed version, considering all aspects mentioned in the original report. The expanded version should be at least 4 sentences long. Ensure that you elaborate on each point from the original report, providing more context and explanation where possible. Do not add any new content, interpretations, or conclusions beyond what is explicitly stated in the original report. Avoid using phrases like "Here is the revised report" or similar introductions. Simply begin with the expanded content.

Original Report:
{report}
Expanded Report:

Supplementary Figure 7: The prompt used to revise (and translate) original reports. Figure created with PowerPoint.

Prompt: Instruction Data Scoring

Task: Given an ECG report and a corresponding question-answer pair, score the quality of the answer based on the guidelines provided. The score should range from 0 to 5, where 0 represents poor quality and 5 represents excellent quality. You should be strict when giving the final assessment if some of the criteria are not satisfied. Please consider the following criteria for scoring:

1. Relevance: Does the answer directly address the question asked?
2. Accuracy: Is the information in the answer accurate and consistent with the ECG report?
3. Usefulness: Does the answer provide helpful information that would aid understanding or decision-making based on the ECG report?
4. Constructed Information: Does the answer invent details not present in the ECG report?
5. Presence of Direct Report Quotation: A good answer does not simply quote or directly replicate phrases from the ECG report. It should assume that the questioner does not know the report's specific content. The presence of direct report quotations is not allowed in the answer, otherwise, the overall scores should be at most 2.

Output format:
Please first output a single line containing a comprehensive explanation of your evaluation, avoiding any potential bias. In the subsequent line, please provide the value indicating the scores in the format: "Score: [your rating score]"

Please apply the above scoring guide to the following ECG report and question-answer pair:

ECG Report: {report}
Question: {question}
Answer: {answer}

Supplementary Figure 8: The prompt used to score and filter generated instruction data. Figure created with PowerPoint.

Prompt: Evaluation of Report Generation
Evaluate the alignment and quality of a generated ECG report by comparing it to a ground truth clinician's report. The evaluation will focus on three key aspects: Diagnosis, Waveform, and Rhythm. Use specific criteria for each aspect and be precise in comparing medical terminologies. Only focus on information present in the ground truth report, identifying any mistakes. Remain objective and do not let the response length affect your evaluation.<br><br>Evaluation Criteria:<br>**1. Diagnosis (0-10):**<br>Assess how well the generated ECG report matches the clinical diagnoses in the ground truth report. Focus on conditions like conduction disturbances, ischemia, hypertrophy, and other abnormalities as presented in the ground truth report.<br>- 10: All key diagnoses are correctly identified with no errors or omissions.<br>- 5: Partially accurate, with some diagnoses identified correctly but key conditions missing or incorrect.<br>- 0: Fails to identify key diagnoses, with multiple critical errors.<br><br>**2. Waveform (0-10):**<br>Evaluate the accuracy and quality of the ECG waveform morphology in the generated report compared to the ground truth. Focus on abnormalities in P-wave, QRS complex, ST changes, T-wave, and intervals (PR, QT), ensuring waveform morphology is consistent with the ground truth.<br>- 10: All waveform abnormalities are correctly identified without errors.<br>- 5: Some waveform abnormalities are identified, but key issues are missed or misinterpreted.<br>- 0: Fails to identify key waveform abnormalities, with multiple critical errors.<br><br>**3. Rhythm (0-10):**<br>Assess the accuracy and clarity of rhythm interpretation in the generated report. Focus on identifying and describing normal and abnormal rhythms (e.g., sinus rhythm, atrial fibrillation, ventricular tachycardia) as presented in the ground truth report.<br>- 10: Rhythm interpretation is fully accurate and clearly described.<br>- 5: Rhythm interpretation is partially accurate but contains notable errors or omissions.<br>- 0: Rhythm interpretation is largely incorrect, with critical errors.<br><br>Please organize your output in a JSON format of diagnosis, form and rhythm, with a brief explanation of each aspect. For example: {Diagnosis: {Score: $SCORE$, Explanation: $EXPLANATION$}}<br><br>[The Start of Ground Truth Report]<br>{ground_truth_report}<br>[The End of Ground Truth Report]<br>[The Start of Generated Report]<br>{generated_report}<br>[The End of Generated Report]

Supplementary Figure 9: The prompt used to evaluate the generated report. Figure created with PowerPoint.

Prompt: Evaluation of ECG Arena

Evaluate the quality of a model's response to an ECG-related question by comparing it with a given ground truth answer. Focus on three aspects: accuracy, completeness, and instruction adherence. Be precise and objective, especially when identifying errors in medical terminology. Do not let the response length affect your evaluation.

Evaluation Criteria:
1. Accuracy (0-10):
How well does the model's response match the ground truth, particularly in ECG interpretation and diagnosis? This score emphasizes whether the key information is correct, such as the correct identification of waveforms, intervals, and clinical diagnoses.
- 10: Fully accurate, with correct ECG interpretation, terminology, and diagnosis.
- 5: Partially accurate, with some correct information but notable errors or omissions.
- 0: Largely inaccurate or misleading.

2. Completeness (0-10):
Does the response cover essential aspects of ECG interpretation (e.g., rhythm, axis, waveforms, clinical causes) mentioned in the ground truth? This score focuses on whether the answer is comprehensive and includes as much essential information as possible.
- 10: Comprehensive, covering all key details.
- 5: Partially complete, with important points missing.
- 0: Incomplete, lacking critical information.

3. Instruction Adherence (0-10):
Does the model follow the specific instructions in the question (e.g., listing features, suggesting a diagnosis)? This score focuses on how well the model follows the task instructions, regardless of the correctness of the answer.
- 10: Fully follows instructions.
- 5: Partially follows instructions, with some deviations.
- 0: Fails to follow instructions or provides an irrelevant response.

Please organize your output in a JSON format of accuracy, completeness, and instruction adherence, with a brief explanation of each aspect. For example: {Accuracy: {Score: $SCORE$, Explanation: $EXPLANATION$}}

[The Start of Ground Truth Answer]
{ground_truth_answer}
[The End of Ground Truth Answer]

[The Start of Model's Response]
{model_response}
[The End of Model's Response

Supplementary Figure 10: The prompt used to evaluate the ECG Arena. Figure created with PowerPoint.

C Main Results

We provide the overall results in Table 3 for in-domain evaluation and Table 4 for out-of-domain evaluation.

Datasets	PTB-XL Super			PTB-XL Report	CODE-15%			ECG-QA
Metric	AUC	F1	HL	Report Score	AUC	F1	HL	Accuracy
Random	50.3	33.2	50.1	0	48.8	15.0	32.1	16.2
Domain-specific Methods								
METS	-	65.7†	-	N/A	-	-	-	N/A
MERL	74.2†	-	-	N/A	-	-	-	N/A
ST-MEM	71.4†	-	-	N/A	-	-	-	N/A
MMCL	81.6	-	-	N/A	-	-	-	N/A
MOMENT	**83.3**	-	-	N/A	-	-	-	N/A
ECG-GPT	69.5*	53.9*	20.1*	47.8*	68.9*	40.1*	17.4*	N/A
Proprietary MLLMs								
GPT-4o	55.6	28.3	26.2	50.2	59.9	24.9	15.7	35.2
GPT-4o mini	52.0	20.4	31.7	37.1	57.5	22.0	15.1	14.9
Gemini 1.5 Pro	50.7	15.3	27.9	35.9	56.7	20.0	15.9	33.2
Claude 3.5 Sonnet	54.0	27.5	29.6	43.7	58.3	20.3	17.8	34.2
Open-source MLLMs								
LLaVA-Med	50.0	12.3	28.1	24.3	69.2	27.0	33.4	29.5
LLaVA-1.5-7B	50.0	12.3	28.1	27.2	63.9	19.2	25.3	25.2
LLaVA-1.5-13B	50.0	35.2	48.4	20.7	53.9	13.1	13.6	21.2
LLaVA-1.6-Vicuna-7B	50.0	15.8	29.4	16.5	50.1	1.0	13.6	13.3
LLaVA-1.6-Vicuna-13B	50.0	20.1	38.3	5.9	53.0	3.6	16.6	22.0
LLaVA-1.6-34B	50.2	19.9	36.0	17.0	57.2	12.8	16.6	22.4
LLaVA-OneVision-7B	49.8	11.4	34.5	30.0	58.7	17.0	20.6	20.4
LLaVA-OneVision-72B	50.6	29.6	50.4	40.6	52.3	7.0	13.1	25.0
Deepseek-VL-Chat-7B	50.9	15.7	27.9	15.6	63.7	27.5	22.4	21.1
Idefics2-8B	50.7	21.9	31.2	10.6	49.0	17.9	47.9	26.1
Mantis-8B-siglip-Llama3	50.6	20.4	30.0	16.0	57.5	17.9	15.7	23.8
MiniCPM-V-2.6	49.0	37.7	63.8	15.4	56.6	25.3	22.0	20.8
Phi-3-Vision-128k-Instruct	50.0	29.6	48.4	20.2	69.6	22.6	38.8	28.4
Qwen2-VL-7B	51.3	22.4	30.8	43.0	60.7	24.8	20.5	20.4
Qwen2-VL-72B	54.0	28.3	30.2	48.9	60.6	23.6	16.1	23.7
InternVL2-8B	50.6	14.3	27.8	38.1	55.8	16.1	17.7	22.3
InternVL2-40B	51.2	18.7	34.6	41.8	56.7	16.2	17.4	18.2
InternVL2-Llama3-76B	50.4	9.4	35.6	41.4	59.0	20.2	20.5	21.8
PULSE-7B (Ours)	**82.9**	**76.9**	**10.2**	**65.4**	**91.7**	**87.0**	**4.6**	**71.6**
Δ over best proprietary MLLM	+27	+49	+16	+15	+32	+62	+11	+36
Δ over best open-source MLLM	+29	+39	+18	+17	+22	+60	+9	+42

Supplementary Table 3: In-domain evaluation results on representative baselines. † indicates results from original papers, * denotes results obtained using the provided online software, N/A indicates methods not applicable or not designed for certain tasks, and - indicates unreported scores in original papers.

Datasets	CPSC 2018			CSN	G12EC	MMMU ECG	ECG Arena
Metric	AUC	F1	HL	Accuracy	Accuracy	Accuracy	Arena Score
Random	51.2	15.1	28.8	11.6	12.1	24.2	0
Domain-specific Methods							
METS	-	-	-	N/A	N/A	N/A	N/A
MERL	**82.8**†	-	-	N/A	N/A	N/A	N/A
ST-MEM	70.4†	-	-	N/A	N/A	N/A	N/A
MMCL	52.7	-	-	N/A	N/A	N/A	N/A
MOMENT	50.5	-	-	N/A	N/A	N/A	N/A
ECG-GPT	69.3*	44.0*	9.9*	N/A	N/A	N/A	N/A
Proprietary MLLMs							
GPT-4o	50.9	10.6	18.2	57.5	49.2	43.5	33.5
GPT-4o mini	49.2	11.0	25.5	32.1	33.2	39.5	30.1
Gemini-1.5-Pro	50.1	7.4	20.5	50.5	36.0	40.0	31.2
Claude 3.5 Sonnet	52.8	11.5	18.9	51.5	51.4	42.0	37.1
Open-source MLLMs							
LLaVA-Med	50.0	2.5	20.2	13.8	14.1	27.0	15.9
LLaVA-1.5-7B	50.0	2.5	20.0	32.1	25.4	33.0	12.7
LLaVA-1.5-13B	50.4	13.3	30.1	30.7	30.7	35.0	13.1
LLaVA-1.6-Vicuna-7B	50.5	19.7	66.0	23.7	23.3	28.0	16.0
LLaVA-1.6-Vicuna-13B	50.0	19.3	62.8	31.4	35.0	38.0	17.9
LLaVA-1.6-34B	49.6	19.3	62.8	44.3	45.9	31.0	17.5
LLaVA-OneVision-7B	49.6	8.0	28.3	23.3	25.7	26.0	22.5
LLaVA-OneVision-72B	51.5	12.8	29.4	44.0	42.6	35.0	15.5
Deepseek-VL-Chat-7B	50.7	6.0	20.0	35.7	32.9	34.5	15.3
Idefics2-8B	49.0	17.9	47.9	22.8	26.2	36.0	4.9
Mantis-8B-siglip-Llama3	51.3	19.1	48.5	17.6	22.6	38.5	13.6
MiniCPM-2.6	50.0	18.0	48.4	12.7	19.6	34.5	20.4
Phi-3-Vision-128k-Instruct	50.6	19.0	70.2	14.8	18.4	31.0	11.3
Qwen2-VL-7B	49.4	17.5	46.3	25.5	32.9	31.5	8.5
Qwen2-VL-72B	50.7	9.8	18.9	35.5	42.9	35.0	10.3
InternVL2-8B	52.1	8.2	22.2	47.7	37.5	30.0	22.9
InternVL2-40B	52.4	8.2	21.4	41.0	45.0	30.5	28.0
InternVL2-Llama3-76B	51.3	6.5	20.4	26.6	34.7	38.0	22.5
PULSE-7B (Ours)	80.7	**65.4**	**6.8**	**87.9**	**81.4**	**64.0**	**41.5**
Δ over best proprietary MLLM	+28	+54	+11	+30	+30	+21	+4
Δ over best open-source MLLM	+28	+46	+12	+40	+36	+26	+14

Supplementary Table 4: Out-of-domain evaluation results. † indicates results from original papers, * denotes results obtained using the provided online software, N/A indicates methods not applicable or not designed for certain tasks, and - indicates unreported scores in original papers.

D Ablation Study

Effect of Freezing the Vision Encoder. We performed an ablation study by freezing the vision encoder parameters during training and reported the model's performance in Table 5. The results indicate a further decline in performance, with the average score dropping from 71.2 to 68.1, compared to the original model with unfrozen vision encoder parameters.

Models	PTB-XL Super	PTB-XL Report	CSN	CODE-15	ECG-QA	CPSC	G12	MMMU ECG	ECG Arena	**AVG**
Unfrozen ViT	**76.9**	**65.4**	**87.9**	**87.0**	71.6	**65.4**	**81.4**	**64.0**	**41.5**	**71.2**
Frozen ViT	74.8	61.3	85.2	85.4	**73.8**	57.6	78.2	58.0	38.9	68.1

Supplementary Table 5: Ablation results on the impact of freezing vision encoder parameters

Fine-tuning results against other MLLMs We fine-tune Qwen2-VL-7B using `ECGInstruct` and show the results in Table 6. The performance of the two backbone models is comparable, with LLaVA slightly outperforming Qwen.

Models	PTB-XL Super	PTB-XL Report	CSN	G12	MMMU ECG
PULSE (Qwen2-VL-7B)	75.1	62.8	85.2	79.2	60.4
PULSE (LLaVA-v1.6 -Vicuna-7B)	**76.9**	**65.4**	**87.9**	**81.4**	**64.0**

Supplementary Table 6: Comparison of different MLLM backbones

Effect of Instruction Task. To understand the individual contribution of each ECG-related task to model performance, we analyze combinations of four instruction tasks. As shown in Table 7, adding more tasks progressively improves performance across multiple benchmarks. Models trained solely on basic feature recognition (F) performed poorly across all metrics, highlighting the limitations of a single-task approach. In contrast, the sequential addition of tasks led to substantial performance gains across multiple benchmarks. The model incorporating all four tasks achieved the highest performance, indicating a more comprehensive understanding of ECG images.

Instruction Task	PTB-XL Super	PTB-XL Report	CSN	CODE-15	ECG-QA	CPSC	G12	MMMU ECG	ECG Arena	**AVG**
F	14.4	40.1	59.3	12.8	52.6	10.3	14.4	40.0	15.0	28.8
F + R	29.0	58.1	86.5	74.9	59.2	38.8	70.5	53.5	27.9	55.4
F + R + M	72.5	61.7	87.9	84.3	66.4	51.6	74.2	58.5	33.0	65.6
F + R + M + C	76.9	65.4	87.9	87.0	71.6	65.4	81.4	64.0	41.5	**71.2**

Supplementary Table 7: Performance of different ECG-related instruction task combinations. F: basic feature recognition, R: heart rhythm analysis, M: morphology and pathology identification, C: clinical report generation. F1 for PTB-XL Super, CODE-15%, and CPSC; Accuracy for CSN, ECG-QA, G12, and MMMU ECG; Report Scores for PTB-XL Report; Arena Scores for ECG Arena. **AVG** denotes the average across all metrics.

E Human Expert Performance

Participant ID	Gender	Department	Job Title	Years of Experience
Expert A	Female	Cardiology	Physician	12
Expert B	Male	Cardiology	Senior Resident	5
Expert C	Male	Cardiology	Physician	15

Supplementary Table 8: Demographic and professional information of participating human experts in the MMMU ECG evaluation.

F Human Evaluation

We performed a human evaluation on 50 sampled reports from the PTB-XL Report and 30 questions from the ECG Arena. Specifically, two independent cardiologists are involved to validate the model-generated answers with the same rubrics. Human evaluation scores and their Pearson correlation with LLM-based scores are presented in Table 9. The results indicate a strong correlation between the two sets of scores.

Models	PTB-XL Report (LLM score)	PTB-XL Report (Human score)	Pearsons Correlation	ECG Arena (LLM score)	ECG Arena (Human score)	Pearsons Correlation
GPT-4o	51.9	50.8	93.4	32.8	35.1	92.0
PULSE	62.8	64.1	91.9	37.4	39.0	91.7

Supplementary Table 9: Comparison of human evaluation scores and LLM-based scores on report generation and ECG arena.

G Case Study

We provide three examples of ECG report generation (Fig. 11, Fig. 12) and one example of ECG Arena (Fig. 13).

Case Study: PTB-XL Report

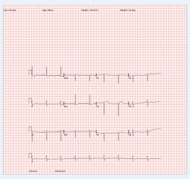

Question:
Please write a clinical report based on this ECG image.

Ground Truth Report:
Sinus rhythm. Prolonged PR interval. R-S transition zone in V leads displaced to the left. Voltages are high in limb leads suggesting left ventricular hypertrophy (LVH). ST segments are depressed in leads I, II, aVL, V5, and V6. T waves are flat in these leads. Non-specific but consistent with myocardial changes.

PULSE Response: The electrocardiogram (ECG) reveals a **sinus rhythm**, indicating that the heart is beating in a regular, rhythmic pattern, with a normal heart rate and P-wave morphology. **The PR interval is prolonged**, measuring longer than the normal range of 120-200 milliseconds, suggesting a delay in the conduction of electrical impulses from the atria to the ventricles. This prolongation may be indicative of a first-degree atrioventricular (AV) block, which can be a benign finding but may also be associated with underlying cardiac conditions. The ECG also demonstrates **high voltages in the limb leads**, specifically leads I, II, and III, which is **suggestive of left ventricular hypertrophy (LVH).** This finding is consistent with an increase in the thickness of the left ventricular wall, which can be a response to chronic pressure overload or volume overload. **The ST segments are depressed in leads I, II, aVL, V5, and V6**, indicating a possible alteration in the repolarization pattern of the ventricles. This depression may be indicative of left ventricular strain or ischemia, which can be a sign of myocardial injury or infarction. Furthermore, the **T waves are low or flat in leads I, II, aVL, V5, and V6**, which may be a manifestation of left ventricular strain or ischemia. The combination of these findings, including the prolonged PR interval, high voltages in the limb leads, and ST segment depression, **suggests that the patient may be experiencing myocardial ischemia**, which is a condition where the heart muscle does not receive sufficient blood flow, leading to a mismatch between oxygen demand and supply. In conclusion, based on the ECG findings, the diagnosis is consistent with **myocardial ischemia, left ventricular hypertrophy, T wave abnormality, prolonged PR interval, and sinus rhythm**.

LLM-as-Judge Feedback for PULSE Response:
1. Diagnosis
Score: 10. Explanation: The generated report correctly identifies all key diagnoses present in the ground truth report, including sinus rhythm, prolonged PR interval, left ventricular hypertrophy (LVH), ST segment depression, and T wave abnormalities. The interpretation of these findings as consistent with myocardial ischemia is also accurate.
2. Form
Score: 10. Explanation: The generated report accurately describes the waveform abnormalities, including the prolonged PR interval, high voltages in the limb leads, ST segment depression in leads I, II, aVL, V5, and V6, and flat T waves in these leads. These descriptions match the ground truth report precisely.
3. Rhythm
Score: 10. Explanation: The generated report correctly identifies the rhythm as sinus rhythm, which is consistent with the ground truth report. The description of the rhythm is clear and accurate.
Final Score: (10+10+10)/3 * 10 = 100

GPT-4o Response:
Clinical Report: Electrocardiogram (ECG)
ECG Interpretation: Heart Rate: The heart rate appears to be within normal limits. Precise calculation of the heart rate is recommended for confirmation. Rhythm: The rhythm appears to be regular. There are consistent R-R intervals, suggesting a **regular sinus rhythm**.
P Waves: P waves are present before each QRS complex, indicating sinus rhythm. The morphology of the P waves appears normal. **PR Interval: The PR interval appears to be within normal limits (120-200 ms).** QRS Complex: The QRS duration appears to be within normal limits (less than 120 ms). There is no evidence of significant QRS complex abnormalities such as bundle branch block.
ST Segment: **The ST segments appear to be isoelectric (flat) in most leads, with no significant elevation or depression noted**. **T Waves: T waves appear to be upright in leads I, II, V3-V6, and inverted in aVR, which is normal. There are no significant T wave abnormalities noted**. Axis: The QRS axis appears to be normal. The exact axis calculation is recommended for confirmation.
Additional Observations: There are **no significant signs of ischemia, infarction, or other acute changes**. **No evidence of left ventricular hypertrophy or other chamber enlargement**.
Conclusion: This ECG shows a normal sinus rhythm **with no significant abnormalities. The patient's ECG is within normal limits for her age and clinical presentation.**

LLM-as-Judge Feedback for GPT-4o Response:
1. Diagnosis
Score: 0. Explanation: The generated report fails to identify key diagnoses present in the ground truth report. It does not mention the prolonged PR interval, left ventricular hypertrophy (LVH), ST segment depression, or flat T waves. Instead, it incorrectly states that there are no significant signs of ischemia, infarction, or other acute changes, and no evidence of left ventricular hypertrophy.
2. Form
Score: 0. Explanation: The generated report inaccurately describes the waveform morphology. It incorrectly states that the PR interval is within normal limits, fails to identify the high voltages in limb leads, and does not mention the ST segment depression or flat T waves. These are critical waveform abnormalities that are present in the ground truth report.
3. Rhythm
Score: 5. Explanation: The generated report correctly identifies a regular sinus rhythm, which is consistent with the ground truth report. However, it fails to mention the prolonged PR interval, which is a notable omission. Therefore, while the rhythm interpretation is partially accurate, it contains a significant error.
Final Score: (0+0+5)/3 * 10 = 16.7

Supplementary Figure 11: Comparison of model outputs on ECG report generation task (Example 1). Blue indicates correct information, while red highlights errors. Our model's output fully aligns with the ground truth, with a report score of 10. In comparison, GPT-4's report, though structurally sound, contains notable inaccuracies despite its initial appearance of relevance. Figure created with PowerPoint.

Case Study: PTB-XL Report

Question:
Please write a clinical report based on this ECG image.

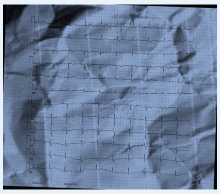

Ground Truth Report:
Sinus rhythm. T waves are low or flat in V3, V4, V5 and slightly inverted in V2. Non-specific but consistent with myocardial ischemia. The age of the changes is uncertain.

PULSE Response: The electrocardiogram (ECG) reveals a **normal sinus rhythm**, characterized by a regular heartbeat with a rate between 60-100 beats per minute, indicating that the heart's natural pacemaker, the sinoatrial (SA) node, is functioning properly. The axis of the heart's electrical activity is also within normal limits, suggesting that the electrical impulses are being conducted through the heart in a normal and coordinated manner. However, the ST segment and **T waves exhibit abnormalities, which may be indicative of myocardial ischemia or strain**, particularly in the anterior region of the heart. This could be a sign of reduced blood flow to the heart muscle, potentially leading to damage or even necrosis of the myocardial tissue. The presence of these abnormalities in the ST segment and T waves suggests that the heart is under stress, which may be caused by a variety of factors, including coronary artery disease, hypertension, or cardiomyopathy. Overall, the diagnosis based on this ECG report is **sinus rhythm** with ST changes, indicating that the heart is functioning normally in terms of rhythm, but is **exhibiting signs of potential myocardial ischemia or strain**.

LLM-as-Judge Feedback for PULSE Response:
1. Diagnosis
Score: 8. Explanation: The generated report correctly identifies the sinus rhythm and the presence of abnormalities in the ST segment and T waves, which are consistent with myocardial ischemia. However, it does not specifically mention the low or flat T waves in V3, V4, V5, and the slightly inverted T wave in V2, which are key details in the ground truth report.
2. Form
Score: 7. Explanation: The generated report accurately identifies the presence of ST segment and T wave abnormalities, which are indicative of myocardial ischemia. However, it lacks the specific details about the T wave morphology in V2, V3, V4, and V5, which are crucial for a precise comparison with the ground truth report.
3. Rhythm
Score: 10. Explanation: The generated report correctly identifies the sinus rhythm and provides a clear and accurate description of the heart's rhythm, matching the ground truth report.
Final Score: (8+7+10)/3 * 10 = 83.3

GPT-4o Response:
ECG Interpretation:
1. Heart Rate: The heart rate is approximately 75 beats per minute, which is within the normal range (60-100 bpm).
2. Rhythm: The rhythm appears to be regular with consistent R-R intervals, indicating a **normal sinus rhythm**.
3. P Waves: P waves are present before each QRS complex, indicating normal atrial depolarization. The P wave morphology is normal.
4. PR Interval: The PR interval is within the normal range (120-200 ms), suggesting normal atrioventricular conduction.
5. QRS Complex: The QRS duration is within the normal range (less than 120 ms), indicating normal ventricular depolarization. There are no significant Q waves observed, which rules out previous myocardial infarction.
6. ST Segment: The ST segments are isoelectric (flat) in all leads, indicating no signs of acute ischemia or injury.
7. T Waves: **T waves are upright in leads I, II, V3-V6, and inverted in aVR, which is a normal finding. There are no signs of T wave inversion or hyperacute T waves.**
8. QT Interval: The QT interval appears to be within the normal range (less than 440 ms for males and less than 460 ms for females), indicating no prolonged QT syndrome.
9. Axis: The QRS axis is normal, approximately 0 to +90 degrees.
10. Additional Observations: No evidence of left or right ventricular hypertrophy. No signs of bundle branch block or other conduction abnormalities.
Conclusion: The ECG findings are within normal limits. There are **no signs of acute ischemia, infarction**, or other significant cardiac abnormalities. The patient has a **normal sinus rhythm** with normal atrioventricular and intraventricular conduction.

LLM-as-Judge Feedback for GPT-4o Response:
1. Diagnosis
Score: 0. Explanation: The generated report fails to identify the key diagnosis of myocardial ischemia indicated by the T wave abnormalities in the ground truth report. It incorrectly concludes that there are no signs of acute ischemia or other significant cardiac abnormalities.
2. Form
Score: 0. Explanation: The generated report does not mention the low or flat T waves in V3, V4, V5, and the slightly inverted T waves in V2, which are critical waveform abnormalities noted in the ground truth report. Instead, it incorrectly states that T waves are upright in V3-V6 and does not acknowledge any T wave inversion.
3. Rhythm
Score: 10. Explanation: The generated report correctly identifies the rhythm as normal sinus rhythm, which is consistent with the ground truth report.
Final Score: (0+0+10)/3 * 10 = 33.3

Supplementary Figure 12: Comparison of model outputs on ECG report generation task (Example 2). Blue indicates correct information, while red highlights errors. Our model's output mostly aligns with the ground truth, achieving a report score of 83.3. In comparison, GPT-4's output correctly identifies only the ECG rhythm, omitting most other key details. Figure created with PowerPoint.

Case Study: ECG Arena

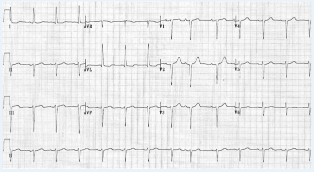

Question:
What can be inferred from the pattern of the qR complexes and rS complexes in the leads of this ECG image?
Ground Truth Answer:
The pattern of qR complexes in leads I and aVL, along with rS complexes in leads II, III, and aVF, is characteristic of a Left Anterior Fascicular Block (LAFB). In LAFB, the ECG typically shows small Q waves and tall R waves in leads I and aVL (qR complexes), and small R waves followed by deep S waves in leads II, III, and aVF (rS complexes). This pattern is a result of the altered conduction through the left anterior fascicle, which causes the electrical activity to spread upwards and leftwards in the heart, leading to these distinctive ECG findings.
Follow-up Question:
What is the key feature that supports the diagnosis in this ECG image?
Ground Truth Answer:
The left axis deviation (LAD) observed in the ECG is a key feature that supports the diagnosis of Left Anterior Fascicular Block (LAFB). LAD is indicated by the negative deflections in leads II, III, and aVF, and positive deflections in leads I and aVL. In LAFB, the abnormal conduction pathway leads to the electrical axis of the heart shifting towards the left, which manifests as a left axis deviation on the ECG. This deviation, combined with the specific patterns of qR complexes in leads I and aVL and rS complexes in leads II, III, and aVF, strongly suggests LAFB.

PULSE Response to the First Question:
The presence of qR complexes and rS complexes in the leads suggests a pattern of abnormal ventricular depolarization. **The qR complexes, which are small q waves followed by a larger R wave, indicate a possible conduction delay or block in the ventricular conduction system**. **The rS complexes, which are small R waves followed by a larger S wave, may indicate a pattern of abnormal ventricular activation, possibly related to the left anterior fascicular block.** The combination of these complexes suggests that the ventricular depolarization is not following a normal pattern, which can be indicative of underlying cardiac pathology.
PULSE Response to the Follow-up Question:
The key feature that supports the diagnosis in this ECG image is the presence of a **left anterior fascicular block**…The left anterior fascicular block is a specific type of conduction block that affects the left anterior fascicle of the left bundle branch, resulting in abnormal ventricular activation. This diagnosis is further supported by the **presence of left ventricular hypertrophy**, which can contribute to the development of conduction abnormalities.

LLM-as-Judge Feedback for PULSE Response:
1. Accuracy
Score: 5. Explanation: The model's response correctly identifies the presence of qR and rS complexes and associates them with a conduction abnormality, specifically a left anterior fascicular block (LAFB). However, it introduces inaccuracies by suggesting a general 'abnormal ventricular depolarization' and mentioning left ventricular hypertrophy, which is not part of the ground truth answer.
2. Completeness
Score: 5. Explanation: The model's response partially covers the essential aspects of ECG interpretation related to LAFB, such as the identification of qR and rS complexes. However, it misses critical details like the specific leads involved (I, aVL, II, III, aVF) and the explanation of left axis deviation (LAD) as a key feature supporting the diagnosis.
3. Instruction Adherence
Score: 7. Explanation: The model follows the instructions by addressing the pattern of qR and rS complexes and suggesting a diagnosis. However, it deviates slightly by introducing unrelated information about left ventricular hypertrophy and not explicitly mentioning left axis deviation (LAD) as the key feature supporting the diagnosis.
Final Score: (5+5+7)/3 * 10 = 56.7

GPT-4o Response to the First Question:
The ECG image shows a pattern of qR complexes in leads V1 and V2, and rS complexes in leads V5 and V6. **This pattern is indicative of a right bundle branch block (RBBB).** Here are the key features that support this diagnosis:
1. qR Complexes in V1 and V2: In RBBB, the initial part of the QRS complex is normal because the left ventricle is activated first. This is followed by a delayed right ventricular activation, which results in a secondary R wave (R') in the right precordial leads (V1 and V2). This can appear as a qR complex. 2. rS Complexes in V5 and V6: In the left precordial leads (V5 and V6), the delayed right ventricular activation results in a wide S wave….In summary, the pattern of qR complexes in V1 and V2, and rS complexes in V5 and V6, along with a prolonged QRS duration, **is characteristic of a right bundle branch block.**
GPT-4o Response to the Follow-up Question:
The key feature that supports the **diagnosis of right bundle branch block (RBBB)** in this ECG image is the presence of a qR complex in leads V1 and V2 and an rS complex in leads V5 and V6. **Additionally, the QRS complex duration is prolonged, which is another hallmark of RBBB.**
To summarize: qR Complex in V1 and V2: This indicates delayed right ventricular activation…These features collectively support the **diagnosis of right bundle branch block**.

LLM-as-Judge Feedback for GPT-4o Response:
1. Accuracy
Score: 0. Explanation: The model's response is largely inaccurate. It incorrectly identifies the pattern of qR complexes in leads V1 and V2 and rS complexes in leads V5 and V6 as indicative of a right bundle branch block (RBBB). The ground truth specifies that the pattern of qR complexes in leads I and aVL, along with rS complexes in leads II, III, and aVF, is characteristic of a Left Anterior Fascicular Block (LAFB). The model's response does not match the correct identification of waveforms, intervals, and clinical diagnosis.
2. Completeness
Score: 0. Explanation: The model's response partially covers the essential aspects of ECG interpretation related to LAFB, such as the identification of qR and rS complexes. However, it misses critical details like the specific leads involved (I, aVL, II, III, aVF) and the explanation of left axis deviation (LAD) as a key feature supporting the diagnosis.
3. Instruction Adherence
Score: 5. Explanation: The model follows the instructions by addressing the pattern of qR and rS complexes and suggesting a diagnosis. However, it deviates slightly by introducing unrelated information about left ventricular hypertrophy and not explicitly mentioning left axis deviation (LAD) as the key feature supporting the diagnosis.
Final Score: (0+0+5)/3 * 10 = 16.7

Supplementary Figure 13: Comparison of model outputs on ECG Arena (Example 1). Blue indicates correct information, while red highlights errors. Given the challenging nature of this task, our model's output partially aligns with the ground truth, and GPT-4o's output largely deviates from the reference. Figure created with PowerPoint.
